# Proteotranscriptomic Dissection of Breast Cancer T Cell States Identifies CD103+ Tfh-derived Cytotoxic Cells Linked to Immunotherapy Response

**DOI:** 10.21203/rs.3.rs-8394722/v1

**Published:** 2026-01-07

**Authors:** Ghamdan Al-Eryani, Sophie Van Der Leij, Etienne Masle-Farquhar, Chia-Ling Chan, Kate Harvey, Sunny Z. Wu, Dan Roden, Taopeng Wang, John Reeves, Bertrand Z. Yeung, Christopher C. Goodnow, Cindy S. Ma, Charles M. Perou, Nir Hacohen, Aziz M. Al’Khafaji, Mats Nilsson, Joakim Lundeberg, Marcel Batten, Simon Junankar, Alexander Swarbrick

**Affiliations:** 1. Broad Institute of MIT and Harvard, Cambridge, MA, USA; 2. Broad Clinical Labs, Burlington, MA, USA; 3. Cancer Ecosystems Program, Garvan Institute of Medical Research, Darlinghurst, NSW 2010, Australia; 4. St Vincent’s Clinical School, Faculty of Medicine and Health, UNSW Sydney, Kensington, NSW 2052, Australia; 5. Translational Genomics Program, Garvan Institute of Medical Research, Darlinghurst, NSW 2010, Australia; 6. Current affiliation: The Westmead Institute for Medical Research, Westmead NSW 2145, Australia; 7. Current affiliation: Genentech Inc., South San Francisco, CA, USA; 8. Current affiliation: Department of Urology, UCSF; 9. Biolegend, San Diego, CA, USA; 10. Lineberger Comprehensive Cancer Center, University of North Carolina at Chapel Hill, Chapel Hill, NC, USA.; 11. Department of Genetics, University of North Carolina, Chapel Hill, NC, USA.; 12. Science for Life Laboratory, Department of Biochemistry and Biophysics, Stockholm University, 171 65 Solna, Sweden.; 13. Science for Life Laboratory, Department of Gene Technology, KTH Royal Institute of Technology, Solna, Sweden; 14. Current Affiliation: Differentia Bio, South San Francisco, California, 94080, USA; 15. Lead contact

## Abstract

While cancer immunotherapies have primarily focused on activation of cytotoxic CD8 cells, CD4 T cell activity is also associated with survival and immunotherapeutic response in numerous cancers. We applied integrated single-cell RNA sequencing and multiplexed protein epitope profiling to breast cancer samples to resolve the complexity of immune cell states within the tumor microenvironment. This approach enhanced phenotypic resolution, identifying three distinct states within the CD4 T follicular helper-like (Tfh) cell cluster. A CXCR4^high^ progenitor state gave rise to two differentiated states: an IGFL2^high^ subset resembling conventional Tfh cells and localised to B cell-rich lymphoid aggregates, and a CD103+ subset, exhibiting features of tissue residency, exhaustion, and cytotoxicity, which co-localised with tumor foci. CD103+ Tfh-like cells were found to interact with CXCL10+ macrophages through production of CCL chemokines and CSF1. A higher CD103+ Tfh to IGFL2^high^ Tfh ratio, together with the selective clonal expansion of the CD103+ subset, was strongly associated with improved tumour immunity and superior responses to anti-PD-1 checkpoint blockade, surpassing the predictive value of exhausted CD8 T cells. These findings integrate Tfh and CD4 with cytotoxic potential in breast cancer, offering new insight into anti-tumor immunity and response to checkpoint blockade.

## Introduction

Solid tumors constitute a diverse ecosystem of cells, the functions of which impact cancer growth and spread^1^. The mixed efficacy of immune and stromal targeted therapies in cancers reflect the dynamic and variable intercellular relationships engaged by cancer cells to sustain malignancy^2,3^.

Breast cancers are characterised by relatively low mutational load and immunogenicity, however, tumor immune infiltration clearly impacts disease outcome^4–8^. The interplay between tumor infiltrating lymphocytes (TILs) and tumor associated macrophages (TAMs) has been proposed to stratify patient prognosis and response to chemotherapy^9,10^. Although immune checkpoint inhibitors (ICI) targeting PD-1 have a modest effect on survival in breast cancer patients compared to other cancer types, such as melanoma, renal or lung cancer^2,11^, their use in combination with chemotherapy is a new standard of care for high-risk, early-stage and metastatic triple-negative breast cancer (TNBC)^12^.

One of the primary therapeutic mechanisms of anti-PD-1 therapy is through prolonged activity of anti-tumor CD8+PD-1+ T cells, which would otherwise be inhibited by PD-L1/L2 signalling within the tumor microenvironment (TME)^11^. CD4+ T follicular helper (Tfh) cells and their counterparts outside lymphoid organs, T peripheral helper (Tph) cells, are also characterised by high PD1 expression^13^ and in a recent single-cell study, we found Tfh-like cells to be the most abundant PD-1-expressing cluster amongst all TILs^7^. We use the all-encompassing term “Tfh-like” to refer to all cells with gene expression features of Tfh cells (*BCL6+ CXCL13*+ *ICOS+ PD1+*). CD4+ T cells are increasingly being linked with response to checkpoint immunotherapy^14–16^ and Tfh-like TILs and tertiary lymphoid structures (TLS; germinal centre-like microstructures in which B cells receive Tfh help) are strongly associated with favourable prognosis across multiple cancers, including breast cancer^6,17–20^. Tfh cells produce CXCL13^13^, and CXCL13-producing CD4 T-cells have been specifically implicated in survival and response to chemotherapy and immunotherapy^4,8,21–23^. However, the mechanisms by which Tfh-like cells enhance tumour immunity remain uncertain.

High-throughput single-cell RNA sequencing (scRNA-seq) is a powerful platform for characterising the cellular constituents of heterogeneous biological systems such as the TME^24–27^. However, scRNA-seq is limited by the sensitivity of RNA measurements at single-cell resolution, a particular challenge with low-transcript containing cells such as lymphocytes, and RNA expression does not always correlate well with protein expression^28–30^. Cellular indexing of transcriptomes and epitopes by sequencing (CITE-Seq) uses antibodies tagged with DNA, and these antibody-derived tags (ADT) are sequenced during scRNA-seq as surrogates for cell surface protein levels^31,32^. Proteotranscriptomics via CITE-Seq permits the linking of transcriptome information with decades of immunological studies that have dissected immune cell states using protein measurements (generally by flow cytometry). CITE-Seq improves upon scRNA-seq-based cellular profiling^33,34^, however, methods and applications for the integration of modalities and synthesis in clustering analysis are still being explored.

Here, we demonstrate how cellular proteotranscriptomics enhances stratification of cell types within the TME, allowing improved identification of lymphocyte subsets associated with clinical outcomes. We find that cells classified as activated tumor-associated Tfh cells by RNA-seq alone are further resolved into distinct states within the tumor microenvironment, and show that they have discrete effector phenotypes, spatial localization with respect to cancer cells and lymphoid aggregates, and signalling potential with macrophages. Supporting the clinical importance of precise cellular phenotyping, we show that the expression signature of one of the identified Tfh-like cell states (marked by CD103+ expression and a cytotoxicity profile) has a stronger correlation with response to anti-PD-1 therapy than other Tfh-like or CD8+ T-cell subsets, including total CXCL13+ CD4 T-cells.

## Results

### Enhanced phenotyping of the tumor microenvironment through integrated RNA-protein based clustering

To better characterize the native tumor immune microenvironment of human breast cancer, we applied a CITE-Seq panel of 97–157 antibodies (157 in 5 samples, 97 in one; **Table S1**) to 6 breast cancer samples, representing all major clinical subtypes: Luminal (estrogen receptor-positive (ER+) and progesterone receptor -positive/negative (PR+/−), human epidermal growth factor receptor positive (HER2+) and Triple negative breast cancer (TNBC) (**Table S2**). 16,423 cells passed the quality filter threshold (Methods; **Figure S1A**), with a variable number of ADT significantly enriched in each sample (MAST test; p_adj < 0.01) and a subset of 32 ADTs common to all samples (**Extended Data Fig. 1B**).

**Figure 1:**
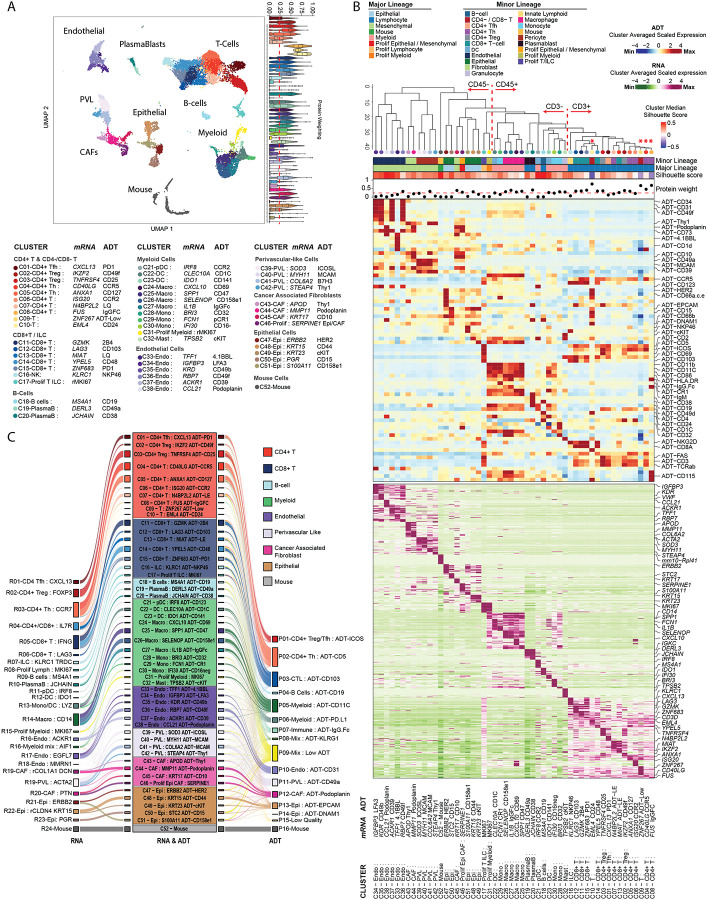
Integrated proteogenomic analysis of the breast tumour microenvironment enhances clustering resolution when compared to ADT or RNA derived clustering. **(A)** UMAP of RNA and ADT integrated clustering analysis identified 52 cell clusters within 6 human breast cancer samples. Violin plots on the right display the relative contribution of ADT markers to the definition of each cluster (“protein weighting”;^33^) and the dashed line marks the median value across all clusters (0.246). **(B)** Differentially enriched ADT (top) and RNA (bottom) features for each cluster (MAST test; p_adj < 0.05), with defining RNA and ADT features labelled on the right. Top annotation dendrogram indicates the cluster relationship derived from integrated RNA and ADT PCA values. Top bar annotations provide the mean silhouette score and median weighted protein weighting for each cluster. **(C)** Alluvial plot visualising the relationship of assigned cell clusters when defined based on RNA alone (left, 27 clusters), integrated RNA and ADT (center, 52 clusters), or ADT alone (right, 16 clusters).

We reasoned that integrated analysis using RNA and ADT data may resolve cellular phenotypes better than single data types. Cells were partitioned using a weighted nearest neighbour (WNN) approach that integrates RNA and surface protein expression^33^, identifying 52 clusters which were labelled based on the most distinctive RNA (*italic*) and ADT-protein (MAST test; p_adj < 0.01) features (Figure 1A–B). The cells captured collapsed into 27 clusters using RNA alone or 16 clusters using ADT data alone for clustering (Figure 1C; **Extended Data Fig. 1D-E**). Despite moderate cell numbers and without employing a lineage specific sub-clustering analysis^7,25,35,36^, this strategy allowed us, for example, to differentiate 6 monocyte and 6 macrophage groups: C28-Mono:*AIF1* CD32hi; C29-Mono:*FCN1* CR1; C30-Mono:*IFI30* CD16; C24-Macro:*CXCL10* CD69; C25-Macro:*SSP1* CD47; C26-Macro:*SELENOP* CD158e1 (Figure 1A–B) as opposed to a single transcriptome-based cluster for each (**Extended Data Fig. 1D**). These clusters demarcate prognostically relevant phenotypes such as TREM2-high lipid associated macrophages (LAM) from CXCL10+ Macrophages^7,9^, or inflammatory monocytes with a cDC2-like profile from classical *CD16+* monocytes^7,25^.

Previous studies of the breast cancer TME have identified important T cell and innate lymphoid cell (ILC) populations using fluorescence-based protein expression assays but these have been difficult to identify in scRNA-seq studies^6,24,37–39^. We identified several CD3+ cell clusters in which protein data heavily influenced clustering (elevated protein modality weighting; Figure 1B - asterisk marked) and which showed low silhouette scores, indicative of poor cluster distinction and stability, when calculated using RNA alone^40^ (**Extended Data Fig. 1F-G**). The requirement for integrated analysis to identify such populations emphasises the value of proteotranscriptomics. Given the strong protein modality weighting in clustering of CD3+ cells, we performed a targeted analysis of T cells.

### Enhanced definition of tumor-infiltrating lymphocytes by targeted CITE-Seq analysis

**Figure 2:**
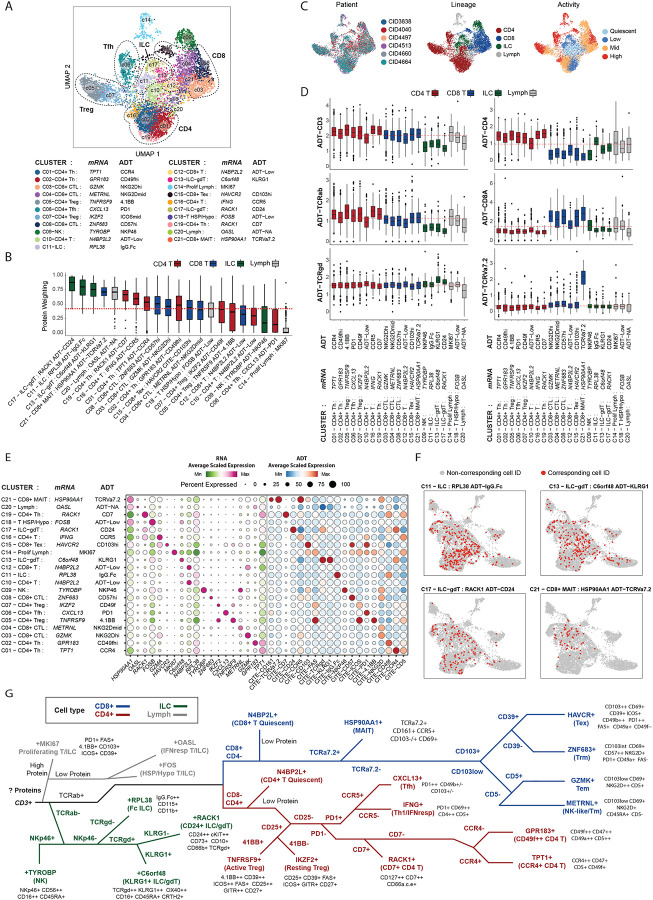
Targeted proteogenomic analysis of T and innate lymphocyte cells improves characterisation of TILs and identifies cell types which cannot be identified by RNA or protein modalities alone. **(A)** UMAP visualisation of integrated clustering of T and innate lymphoid cells derived from 6 human breast cancer samples. Clusters are numbered in order of decreasing cell number. **(B)** Protein weighting score, indicating the weighted proportion of ADT-derived features which contributed to the nearest neighbour calculation for the partitioning of cells into clusters. Increasing scores indicate increasing ‘weight’ of protein markers in the definition of a given cluster. Dashed red line indicates the median protein weighting score across clusters (0.451). Boxplots middle line marks the median value, the lower and upper hinges mark the 25% and 75% quantile, the whiskers correspond to the 1.5 times the interquartile range, the black dot’s mark outliers. **(C)** UMAP plots of cells in (A) colored by the following from left to right: patient sample, broad cell lineage and transcriptional activity score (see Methods). **(D)** Expression of lineage-defining ADT markers across T/ILC clusters, colored by assigned lineage and ordered by cluster size for each respective lineage subset. Boxplots middle line marks the median value, the lower and upper hinges mark the 25% and 75% quantile, the whiskers correspond to the 1.5 times the interquartile range, the black dot’s mark outliers. **(E)** Dotplot of expression (cluster average) of the RNA and ADT markers used to annotate each cluster. **(F)** WNN-derived annotations of clusters C11-ILC, C13-ILC/gdT, C17-ILC/gdT, and C21-CD8+ MAIT cells (red) projected onto the UMAP generated solely by RNA data. **(G)** Protein selection decision tree towards profiling of identified gene expression profile. ‘Low protein’ marks clusters which have low enrichment of ADT-derived UMIs, ‘High protein’ marks cluster which have a high number of ADT-derived UMIs enriched.

A total of 7,515 T/ILC cells were re-clustered using integrated RNA and protein-tag analysis, yielding 21 clusters, each comprising cells from at least three patients (Figure 2A–C; **Extended Data Fig. 2A**). Clusters were first stratified by CD3 and TCRαβ protein expression: high/medium levels indicated conventional or unconventional T cells, while low levels, combined with other markers, identified NK cells or ILCs (Figure 2D–E; **Extended Data Fig. 2B**). Two mixed-expression clusters were labelled as Lymphocytes (C14 & C20). Activity levels (scored from quiescent to high) were assigned using gene signatures^41^, ADT/RNA abundance, ribosomal content^42^, and known activation markers (e.g., CD69, IFNG, GZMB, PD-1,ZNF683/Hobit, CD45RO)^43^ (**Extended Data Fig. 2C**). All CD3+ T cells were segregated based on CD8 or CD4 expression and assessed for expression of unconventional T cell or “NK-like” markers (see Methods; Figure 2D–E; **Extended Data Fig 2B**). Top differentially expressed RNA and protein features for each cluster are shown in Figure 2E and **Table S3**. Published gene signatures (**Table S4**) were used to associate clusters with known lymphocyte functions (**Extended Data Fig. 2D**).

Seven clusters were uniquely identified through integrated analysis that were not distinguishable by analysis of either modality alone (**Extended Data Fig. 2E-G**), including three CD3^low^ TCRαβ^low^ clusters (C11, C13, C17) with resting transcriptional profiles and innate-like protein markers (e.g., KLRG1, OX-40, cKIT, CD112, IgG.Fc), two of which were TCRγδ+ (Figure 2D–E; **Extended Data Fig. 2B**). TCRγδ+ (gamma-delta) T cells express an alternate form of the T cell receptor, recognising non-classical antigens and capable of killing cells without MHC restriction; they have a complex role in cancer^44^. These three clusters lacked contamination, had low mitochondrial and housekeeping gene expression, and minimal ambient/isotype signal (**Extended Data Fig. 2I-J**), suggesting they reflect genuine cell states. MAIT cells (C21), previously linked to anti-tumor immunity^45^, were also resolved and characterized by CD161^high^ TCRVα7.2^high^ expression (Figure 2E). These four clusters had low RNA silhouette scores and high similarity scores, indicating poor resolution in 3’ captured RNA-only analyses (**Extended Data Fig. 2G-H**), as reflected in their dispersion in RNA-only derived UMAPs (Figure 2F), and highlighting the value of CITE-seq.

Integration also improved phenotyping of CD4+ T cell states with low transcriptional activity (Figure 2C; **Extended Data Fig. 2D, F & J**). CD4+ Tregs were stratified into transcriptionally active (C05: *TNFRSF9*^high^4.1BB^high^) and less active (C07: *IKZF2*^high^ ICOS^mid^) states (**Extended Data Fig. 2F**), aligning with profiles previously detected using Smart-seq2^46^, which provides greater gene depth per cell compared to 10X Chromium^47^. CD4+ conventional T cells were further divided into four activation states based on ADT-CD45RO and ADT-CD28, naïve markers such as CCR7 and IL7R and T cell resting enrichment scores (**Extended Data Fig. 2B, C and F**). Integrated clustering also enabled us to separate a transcriptionally quiescent T cell cluster with low CD4 and CD8 transcript levels (C04) into distinct CD4+ (C10) and CD8+ (C12) clusters, also lacking distinguishing protein markers (annotated as ADT-Low; **Extended Data Fig. 2B, C, F**).

Importantly, proteotranscriptomic analysis enables the direct linkage of gene expression signatures derived from scRNA-seq with canonical surface markers historically used to define cellular subsets. For example, CD103 and/or CD69 expression are reported, sometimes interchangeably, as markers of CD8+ tissue resident memory T cells (Trm)^48–50^. However, in our global T cell analysis, we observed CD69 across most T cell and ILC clusters (**Extended Data Fig. 2K**), with increasing expression correlating with RNA signatures of TIL activation, CD48 and CD2 (**Extended Data Fig. 2L**)^51,52^. In contrast, CD103 was more restricted (but not exclusive) to CD8 T-cells, proliferating cells and NK cells (**Extended Data Fig. 2K**) and only modestly correlated with CD69 expression (**Extended Data Fig. 2L**; r=0.29, p =< 0.001) (**Supp Table S5**), except in NK cells (r=0.58, p =< 0.001) and exhausted CD8 T-cells (r=0.59, p = < 0.001). We found the expression of marker 2B4 to correlate most frequently with CD103 across all TILs (r=0.48 p = <0.001) (**Extended Data Fig. 2L**)(**Table S5**).

Among conventional CD8^+^ T cells, we identified four clusters; GZMK+ T-cells (C03: GZMK ADT-NKG2Dhi), METRNL+ T-cells (C04: METRNL NKG2Dmid), ZNF683+ T-cells (C08:ZNF683 ADT-CD57hi) and a cluster often phenotypically described as exhausted (Terminally exhausted T-cells - Tex) or dysfunctional^53^, HAVCR2+ T-cells (C15: HAVCR2 ADT-CD103hi), (**Extended Data Fig. 2D**). Trm cells have been proposed to be marked by CD103 and CD69 expression^54^ in multiple contexts, including studies in human tissues of breast cancers^6^. However, in proteotranscriptomic analysis, C08 ZNF683+ (expressing ZNF683, the primary transcriptional driver of the Trm program^55^) were CD103 intermediate (**Extended Data Fig. 2K**). Instead, Tex cells (C15: HAVCR2 ADT-CD103hi) had the highest expression of CD103 (2-fold higher than C08 ZNF683+ T-cell cluster) (**Extended Data Fig. 2K**). Indeed, analysis of signatures derived from bulk RNA-Seq analysis of FACS-purified human breast cancer TIL subsets (CD103+ CD69+ vs CD103- CD69+)^6,36^ reveals that CD103 and CD69 protein expression primarily marks C15: CD8+ Tex and not C08: ZNF683+ Trm cells (**Extended Data Fig. 2M-N**). Conversely, C08: ZNF683+ cells were more precisely marked by low expression of protein markers CD39 and ICOS, elevated expression of NKG2D and CD57, with only intermediate CD103 expression (**Extended Data Fig. M**; Figure 2E). Taken together, our analysis shows how the direct measurement of both RNA and protein modalities may help enable a more nuanced analysis of T-cells within the TME.

Additionally, we identified CD49f as a robust marker of naive-like and/or recently activated T cells, with its expression consistently decreasing as activation levels increase (as measured using signature markers, ribosome % and CD69) across T cell lineage trajectories (**Extended Data Fig. 2L,P**).

To assist with visualisation of these markers, and provide the field with a guide for flow cytometric panel design, we have created a binary decision tree roadmap using the most distinctive protein markers of each cluster (4 for ILC, 7 for CD4+ T-cells and 6 for CD8+ T-cells), and an array of ADT markers enriched in each cluster (Figure 2G).

### Differentiated subsets within the CD4+CXCL13+PD1+ T follicular helper-like cells can be distinguished by CITE-seq

We previously reported Tfh-like cells to be the most abundant PD-1-expressing cluster amongst all TILs across breast cancer samples^7^, which remained the case in this dataset (Figure 2E, **Extended Data Fig.2B**). Tfh cells acquire distinct phenotypes depending on context^36^￼, and their diversity in breast cancer has been previously alluded to by heterogeneity observed in low-plex fluorescence based assays^18,19,56^. Therefore, we investigated whether enhanced phenotyping by integrated CITE-Seq analysis can assist in dissecting breast cancer Tfh into clinically relevant subtypes.

**Figure 3:**
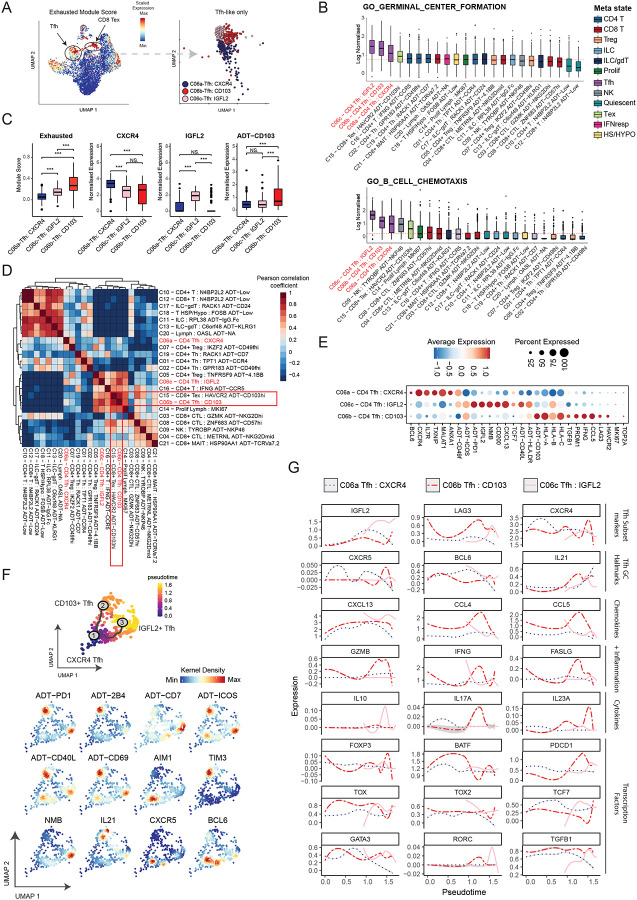
Integrated ADT and RNA clustering reveals novel subsets of Tfh cells in breast cancers. **(A)** Exhausted CD8+ Trm module score overlaid on the T cell/ILC WNN-derived UMAP (see Figure 2A) and UMAP of Tfh subsets derived from WNN of ADT and RNA data **(B)** Gene set enrichment analysis boxplots for the GO biological process pathways “GO_GERMINAL_CENTRE_FORMATION” and “GO_B_CELL_CHEMOTAXIS” across T cells & ILCs, sorted from high to low (left to right). Red line indicates the median expression value across all clusters. Red text marks Tfh populations. Boxplots middle line marks the median value, the lower and upper hinges mark the 25% and 75% quantile, the whiskers correspond to the 1.5 times the interquartile range, the black dot’s mark outliers. **(C)** Tfh cells divided into the 3 identified subclusters are plotted for, from left to right, exhaustion module score, normalised gene expression of *CXCR4* or *IGFL2*, and normalised protein expression of ADT-CD103. A two-sided t-test comparison between each cluster was performed, p-values are denoted by asterisks: *p < 0.05, **p < 0.01, ***p < 0.001 and ****p < 0.0001). Boxplots middle line marks the median value, the lower and upper hinges mark the 25% and 75% quantile, the whiskers correspond to the 1.5 times the interquartile range, the black dot’s mark outliers. **(D)** Pearson correlation heatmap of GO biological process pathways found to be significantly enriched (*n=6614, p* < *0.05*) for each cluster. Red text marks Tfh populations. Red boxes highlight populations of interest outlined in text. **(E)** Dotplot of expression (subcluster average) of the RNA and ADT markers that differentiate the 3 Tfh subclusters. **(F)** UMAP projections of pseudotime analysis using R package *Monocle3*^93^ depicting the bifurcation of CXCR4+ Tfh cells (Node 1) into either the CD103+ exhausted-like state (Node 2) or an IGFL2+ state (Node 3). RNA and ADT density kernel expression of known Tfh-relevant markers overlaid on UMAP projections derived from monocle3 pseudotime analysis. **(G)** Expression of known Tfh-relevant transcription factors, cytokines, chemokines and markers of subset differentiation within this study, stratified by subcluster, along the axis of pseudotime differentiation trajectory.

CD4+ TIL with tumor antigen specificity have been demonstrated to display a PD1+CXCL13+ phenotype and can be divided into distinct functional groups^57^. The C06 cluster in our analysis was defined as PD1+CXCL13+CCR5+ but CD49b+/− and CD103+/− (Figure 2G), indicating a degree of heterogeneity. The C06 phenotype is indicative of Tfh cells but is insufficient to distinguish between Tfh that provide help to B cell in the GC/TLSs and the set of CD4+ TIL with an exhausted, cytotoxic phenotype that have been reported to express a similar set of markers (variously identified as Tph2, cytotoxic Tph, CD4+CTL or CD4+ TCF7-CXCL13+PD1+, Tfh-like cytotoxic CD4+^57–60^). By re-clustering C06 Tfh-like cells, we identified 3 clusters, with the most differentially expressed features annotated: C06a Tfh:CXCR4, C06b Tfh:CD103 and C06c CD4 Tfh: IGFL2 (Figure 3A). All three Tfh-like populations were enriched for expression of genes and pathways associated with Tfh cells (Figure 3B; **Extended Data Fig. 3A-B**).

C06b Tfh:CD103 exhibited a phenotype reminiscent of dysfunctional/exhausted CD8+ T cells^7,53^ (Figure 3C). Indeed, GSEA of GO biological processes (n=6614 pathways, p < 0.05) shows that C06b Tfh:CD103 and C15 CD8 Tex CD103^hi^ exhibited the most similar gene enrichment profiles among all T-cell clusters, even more so than other Tfh-like subsets (Figure 3D, **Extended Data Fig. 3A**).

C06b Tfh:CD103 cells were characterised by upregulation of CD103, 2B4, and CD49b proteins and *LAG3*, *CCL5*, *IFNG, PRDM1* and *HAVCR2* genes, with downregulation of *TCF7* and *BCL6*, relative to the other Tfh-like cell types (Figure 3E, **Extended data Figure 3 B,C**). This profile aligns with that of cytotoxic CD4 cells^59^, Tfh-like cell subsets with cytotoxic features^58,60^ and tumor-specific CD4+ cells with a cytotoxic phenotype identified in melanoma^57^. In contrast, C06c CD4 Tfh: IGFL2 were uniquely marked by elevated expression of the Neuromedin B (*NMB*) and Insulin growth factor ligand 2 (*IGFL2*) genes (Figure 3E). *IGFL2* belongs to a family of 4 genes with unclear biological function that are infrequently and minimally expressed in various tissues, particularly skin, but upregulated during inflammation^61,62^. This C06c subset also expressed the highest levels of CXCL13, relative to other Tfh-like cells (Figure 3E).

Although CXCR5 protein is cleaved from the cell surface during tissue digestion with the enzymatic cocktail mix of Miltenyi Biotech Enzymes A, H and R (**Extended Data Fig. 3D, E**), Tfh-like cells could be identified in flow cytometry using ICOS and PD1 (**Extended Data Fig. 3F)** and interrogated for expression of CD49f, CD103, CD127 and HLA-DR (**Extended Data Fig. 3G**). Our flow cytometric analysis showed differential expression of IL7R, CD40L, PD1, ICOS and HLA-DR between tumor Tfh-like subsets defined based on CD49f and CD103 expression within the CD4+ gate (**Extended Data Fig. 3H**).

The differentiation of Tfh and cytotoxic CD4+ cells has been suggested to branch from a common progenitor^60,63^. We employed pseudotime analysis to determine whether breast cancer Tfh-like cells are transcriptionally related and could be partitioned into stable cell states along a differentiation trajectory. Three stable states (Figure 3 F–G) were identified, suggesting that activated C06a CD4 Tfh:CXCR4 (CXCR4^high^ BCL6^high^ IL7R^high^) can differentiate into either C06b CD103+ Tfh (*HAVCR2*+ *LAG3*+), or C06c IGFL2+ Tfh cells (*NMB*^high^ *CXCL13*^high^) in the TME.

The distinct expression profiles of the segregated Tfh cell subsets suggest that they each have distinct physiological roles within the TME. For example, the C06b CD103+ Tfh cell subset had elevated expression of multiple chemokines including *CCL3*, *CCL4*, and *CCL5*, which are involved in the recruitment of immune cells to the TME^64^, and increased levels of cytotoxicity associated molecules including IFNG, *PRF1*, and *GZMB* (Figure 3E, **Supplementary Table S3**)^36,65,66^. In contrast, the C06c CD4 Tfh:IGFL2 cell population showed comparative upregulation of *IL-10*, *BTLA*, and *CXCL13*, markers important for B cell help and in the maintenance of GC reactions (Figure 3E, G, **Extended data Figure 3B**)^67–70^ and was the cell type most enriched for B cells chemotaxis gene set (Figure 3B). We did not find any of the Tfh-like cell states to express different levels of *FOXP3* (Figure 3G)^19^.

### Tissue CD103+ and IGFL2+ T follicular helper-like cells occupy distinct niches within the tumor microenvironment

**Figure 4:**
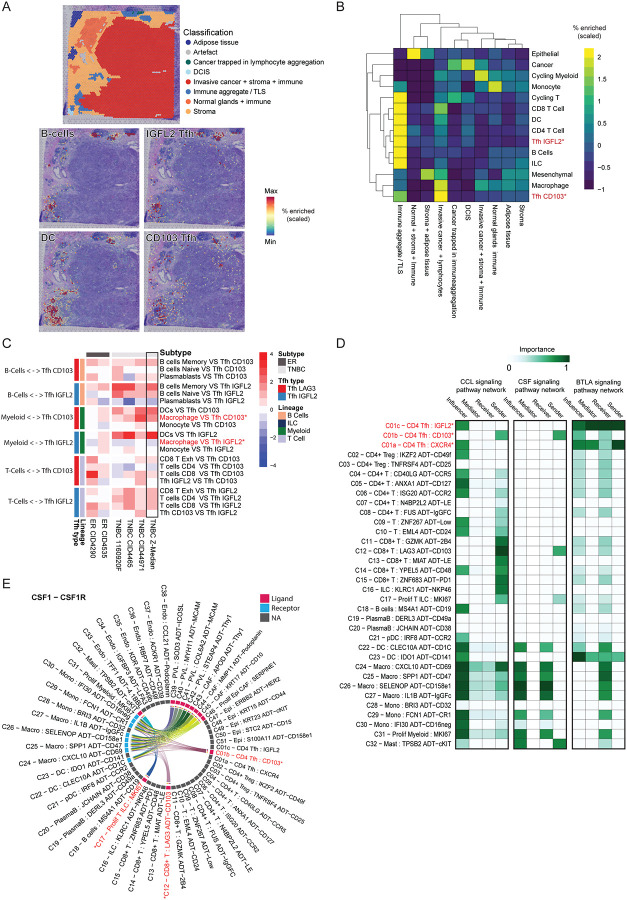
Localisation and signalling of tumour residing CD103+ and IGFL2+ Tfh subsets in breast cancers. Visium spatial transcriptomic analysis **(A)** Enrichment scores for B cells, IGFL2 Tfh (C06c), CD103 Tfh (C06b) and Dendritic cells (DC) overlaid on representative H&E images with pathological annotation shown for reference. **(B)** Heatmap of the enrichment (row scaled) of spatially deconvoluted cell types by pathological annotation as categorised by distinct morphological regions. The median value across 5 breast cancer samples from Wu et al.^7^(3 TNBC, 2 ER+) were used. **(C)** Pearson correlation heatmap of spatially deconvoluted cell pairs of interest. Red text marks populations of interest described in text. **(D)** CCL, CSF and BTLA signalling pathway network characterization across immune cell clusters. ‘Sender’ and ‘Receiver’ status reflects direct expression of ligands and receptors (agonistic or antagonistic). ‘Mediator’ and ‘Influencer’ quantifies the potential role in controlling receptor-ligand expression flow of the pathway within the system (here TME). Red text marks Tfh populations. **(E)** Chord Diagram representing the inferred cell-cell signalling of the CSF1-CSF1R pathway across all immune cells in the dataset. Red text marks populations of interest described in text.

We hypothesised that the Tfh-like cell subsets may reside in unique tissue niches. We assessed data from 5 previously published breast cancer samples (2 ER+, 3 TNBC)^7^ and could delineate the same Tfh-like subsets (**Extended Data Fig. 4A**). We used Visium spatial transcriptomic analysis to identify the location of the two fully differentiated Tfh-like subsets (C06 b and c) and their co-location with other cell types. We observed that C06c Tfh:IGFL2 cell gene signatures are preferentially localised to immune aggregates/structures, as morphologically defined independently by a clinical pathologist (Figure 4 A–B).

Our data showed that C06c CD4 Tfh:IGFL2 cells often co-localise with naive and memory B cells and dendritic cells (Figure 4C; **Extended Data Fig. 4B-C**); C06c IGFL2+ Tfh cells were significantly enriched proximal to CCR7+ CD4 T-cells, B cells and LAMP3+ DCs (mReg: DCs^71^￼), antigen-elicited DC found to engage and regulate tumor reactive T-cells^72^￼ (**Extended Data Fig**. ￼**4D**). In contrast, the C06b Tfh:CD103 cell signature was preferentially localised to regions adjacent to cancer cells (Figure 4 A–B). C06b Tfh:CD103 cells had reduced co-localisation with naive and memory B cells but they had a strong colocalization signal with macrophages (Figure 4C; **Extended Data Fig. 4B-C**). C06b Tfh:CD103 cells were more frequently in proximity to proliferating T-cells and TREM2+ lipid-associated macrophages (**Extended Data Fig**. ￼**4D**), which express elevated PD-L1 protein^7,10^.

The localisation patterns and expression profiles observed support distinct roles for Tfh-like cell states, suggesting that C06c CD4 Tfh:IGFL2 resemble conventional T follicular helper cells, participating in B cell helper functions, whereas C06b Tfh:CD103 are effector, cytotoxic cells that extend into the tumor interface.

Using the expression of cognate receptors and ligands, signalling events and their directionality can be predicted using scRNA-seq data^73^. Our ligand-receptor signalling pathway analysis revealed a number of pathways that were distinct between the C06b and C06c Tfh cell subsets (Figure 4D; **Extended Data Fig. 4 G-G**). Interestingly, the C06b Tfh:CD103 cell subset had a unique chemokine signalling profile reflecting elevated expression of CCL molecules and CSF1 (p < 0.01) (“sender” status, Figure 4E; **Extended Data Figure 4G**). They shared this profile with LAG3+ CD8+ Exhausted T cells which, and together with proliferating T cells, were the only T cell populations expressing CSF1 (Figure 4D–E; **Extended Data Fig. 4G**). Aligning with previous observations, the GO biological processes “Macrophage activation involved in immune response” and “Chronic inflammatory response” were also enriched in this subset (**Extended Data Fig. 4E**), further indicating this cell subset interacts with macrophages within the TME. In contrast, IGFL2+ cells had a significantly elevated predicted signalling of BTLA (Figure 4D, **Extended Data Fig. 4F-G**), an inhibitory receptor associated with conventional Tfh and a pathway that plays a role in regulating GC B cell selection and proliferation^69^.

### Tissue CD103+ Tfh-like helper cells cross-talk with CXCL10 macrophages

**Figure 5:**
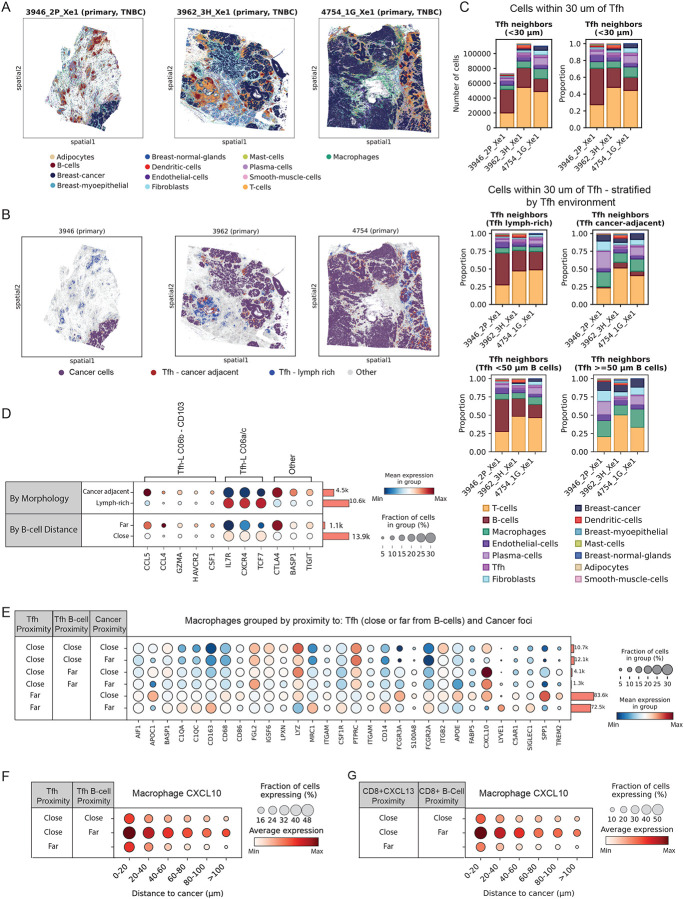
Tissue CD103+ Tfh helper cells cross-talk with CXCL10 macrophages Xenium spatial transcriptomic analysis with the Human Breast 280 plus 100 custom gene panel (Supplementary table S6) was applied to three TNBC samples **(A)** localisation of major cell types as defined by expression profiles according to gene annotation in the Xenium Human Breast panel (3 to 8 gene signatures described in Extended Data 5A). **(B)** Tfh-like cells, as defined by expression of CD4, CXCL13 and BCL6 were categorised based on whether each Tfh had more cancer cells (*Tfh cancer adjacent*, red) or more B cells (*Tfh lymph rich*, blue) within a 200um radiuse. **(C)** Cells categorised based on their spatial location within the tissue in each patient: top panels show cells (annotated with colours, as shown) within 30um of Tfh cells. Middle panels indicate the cells within 30um of the specific cancer-associated or lymph-associated Tfh subsets and the lower panels indicate neighbouring cells of Tfh cells, with panels differentiated by proximity to B cells. **(D)** Expression of key Tfh subset subset marker genes compared between cancer-adjacent Tfh and lymph rich area Tfh; and between Tfh cells proximal or distal to B cells (+/− 50uM). Gene sets relevant to particular Tfh subsets are indicated with brackets. Data presented are the average expression per Tfh group when the cells of the 3 samples are combined. **(E)** CD68+ CD163+ Macrophages were divided into groups on the basis of spatial association with Tfh-like cells, and whether those Tfh-like cells were either *close* (<50u) or *far* or <50um) to B cells or cancer cells. Fraction of cells (circle size) and relative expression (colour) are indicated for a set of 30 macrophage associated genes. **(F)** CXCL10 expression by macrophages at variable distance from cancer cells. 3 types of macrophages were delineated based on proximity within 30um of either B cell associated Tfh or B cell distant Tfh (>50um). **(G)** CXCL10 expression by macrophages at variable distance from cancer cells. 3 types of macrophages were delineated based on proximity within 30um of either B cell associated CD8+CXCL13+ or B cell distant CD8+CXCL13+ (>50um).

Xenium high resolution probe-based spatial transcriptomics was used to investigate the function of each Tfh subcluster at cellular-resolution. We designed custom probe sets targeting TLS and Tfh subset markers **(Supplementary Table S6)**, informed by our scRNA-Seq analysis. Three TNBC whole tissue sections exhibiting distinct tissue morphologies were analysed (Figure 5A). Cells were annotated based on reported lineage markers as annotated in the 10x Human Breast gene panel (See methods) (**Extended Figure 5A**).

Tfh cells were identified by expression of CD4, CXCL13 and BCL6 then categorized based on their spatial location within the tissue, in two analogous ways: (i) by proximity to B cells (**Extended Figure 5B**) and (ii) into cancer-adjacent Tfh cells or Tfh cells in lymph-rich regions based on whether cancer cells or B cells were more common within a 200 μm radius (Figure 5B–C). We assessed the neighbourhoods of either classification and observed that, consistent with our Visium spatial transcriptomic data (Figure 4), Tfh cells adjacent to cancer-cell enriched regions exhibited a marked increase in macrophage co-localization, whereas those located farther away were more commonly associated with B cells (Figure 5B–C; **Extended Data Fig. 5B-C**).

The probes designed to detect IGFL2 and NMB (a key marker of C06c Tfh-like cells) were unfortunately unsuccessful (**Extended Data 5 D-G, Supplementary Table S6**). Therefore, for the purposes of this analysis, TCF7 expression was used to differentiate C06b CD103+ Tfh cells (TCF7-) from grouped C06a CXCR4+ / C06c IGFL2+ Tfh cells (TCF7+) (Figure 3E).

Consistent with our Visium analysis, Xenium gene expression analysis revealed that Tfh-like cells located near tumor regions were enriched for C06b CD103+ Tfh-like signature genes, including CCL5, CCL4, GZMA, HAVCR2, and CSF1. In contrast, Tfh cells residing in lymphoid-rich areas exhibited higher expression of genes associated with C06a/c subsets, such as IL7R, CXCR4, and TCF7 (Figure 5D, **upper; Extended Figure 5E**). When cells were stratified instead by proximity to B cells, Tfh-like cells located distant from B cells (>50um) were enriched for C06b CD103+ Tfh-like signature genes (i.e CCL4, GZMA, HAVCR2) while Tfh cells proximal to B cells exhibited higher expression of genes associated with C06a/c subsets (i.e CXCR4, TCF7) (Figure 5D, **lower; Extended Figure 5D**). These spatial data are consistent with the CD103+ Tfh-like subset not being primarily involved in conventional B cell helper functions (Figure 5D).

We next evaluated changes in the immune cell neighborhood composition based on proximity to Tfh cells, B cells, and the tumor interface. This approach for spatial stratification revealed enrichment of distinct macrophage signatures, including the demarcation of TREM2+SPP1+ lipid-associated macrophages, CXCL10+ inflammatory macrophages, and SIGLEC1+ macrophages^7^ (Figure 5E). Notably, we observed a strong spatial association between cancer proximal Tfh-like cells (analogous to C06b CD103+ Tfh-like cells) and CXCL10+ macrophages (Figure 5E). The CXCL10+ macrophage phenotype was most enriched near these Tfh cells, even as distance from tumor boundaries increased (Figure 5F), suggesting spatially coordinated cross-talk between these populations. A similar spatial association was observed between CXCL10+ macrophages and CD8+ CXCL13+ T cells (Figure 5G), a population we found to exhibit the highest CD103 expression in our dataset (**Extended Data Fig. 2K**). CD8+ CXCL13 T - CXCL10 Macrophage co-localisation has been previously noted in other cancers, including melanoma^74^ colorectal cancer^75^ and lung cancer^76^. Interestingly, we previously identified CXCL10+ macrophages as the highest average PD-L1 expressing macrophage subset^7^, while in this study, we found both Tfh-like cells and CD8+ CD103+ (CXCL13+) T cells to be among the highest PD-1 expressors (**Ext. Data Figure 2P**). These findings, together with the expression similarities observed, suggest a model in which both CD4+ and CD8+ CD103+ T cells may share overlapping functional roles within the tumor microenvironment.

### Tissue CD103+ and IGFL2+ T follicular helper-like ratios are associated with breast cancer subtype and patient prognosis

**Figure 6:**
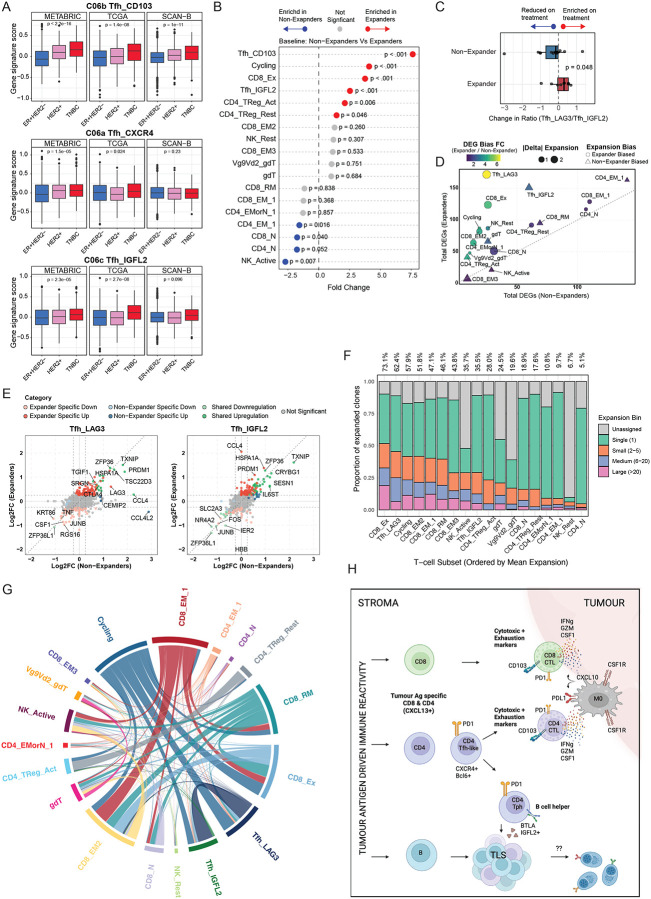
Tfh survival analysis and enrichment analysis of an anti-PD-1 breast cancer cohort. **(A)** Enrichment of gene expression signatures for each Tfh-like subset was quantified in METABRIC^77^, TCGA^78^ and SCAN-B^79^ large breast cancer WTS datasets. Major breast cancer subtypes were partitioned as shown. Boxplots middle line marks the median value, the lower and upper hinges mark the 25% and 75% quantile, the whiskers correspond to the 1.5 times the interquartile range, the black dots mark outliers. ANOVA derived significance values are indicated. **(B)** Cell subsets were defined by expression signature in a cohort of breast cancer patient datasets derived from Bassez et al. (2021)^4^ at the pre-treated time point. The fold change in proportion of each T/NK cell subset was calculated for Expanders (patients with high T-cell clonal expansion after anti-PD1 treatment) relative to Non-expanders (patients with low T clonal expansion after anti-PD1 treatment). P-values calculated from non-parametric t-test (n=29) using package R package Speckle^95^. **(C)** Box plots displaying the patient-matched change (Delta) in the ratio of Tfh_LAG3 (C06b) to Tfh_IGFL2 cells (C06c), calculated individually for each patient (Pre-treatment samples). The dashed line (y=0) denotes no change within a patient. Clinical responders (Expanders, red) exhibit a significant positive shift in the Tfh_LAG3/Tfh_IGFL2 ratio compared to non-responders (Non-Expanders, blue) (P < 0.05, Wilcoxon rank-sum test). **(D)** Scatter plot correlating transcriptional activity with treatment dynamics. Each point represents a T-cell subset. X-axis: Total significant DEGs (p-adjust value < 0.05) in non-expanders pre vs on treatment. Y-axis: Total significant DEGs (p-adjust value < 0.05) in expanders pre vs on treatment. Point Size: Magnitude of the difference in T-cell subset proportion change rates (absolute Delta Expansion) between groups. Fill Color: Ratio of differential gene expression (Expander/Non-Expander). Shape: Direction of expansion bias (Triangle = Non-Expander biased; Circle = Expander biased). The dashed line represents transcriptional parity. **(E)** Scatter plots correlating the treatment log2 fold change of genes in (left) Tfh_LAG3 and (right) Tfh_IGFL2 cells for Expanders (y-axis) vs. Non-Expanders (x-axis). Points represent individual genes. Color: Green = Shared response (significant/concordant in both); Red = Expander-Specific; Blue = Non-Expander-Specific. Dashed lines: Significance thresholds (absolute log2 FC > 0.25, p-adjust value < 0.05). The diagonal indicates perfect concordance. (**F**) Mean expansion frequencies where clonotypes were stratified by cell count into four categories: Single (1), Small (2–5), Medium (6–20), and Large (>20). For normalisation, for each patient and T cell subset, we calculated the proportion of cells in each category and the percentage of all expanded cells (% of T-cells with 2 >= clones). **(G)** Inter-subset clonal relationships as per the Jaccard similarity index was calculated for all pairwise subset combinations within individual patients and the mean repertoire-sharing is visualised, with link width proportional to shared clonotype frequency. **(H)** Schematic summarising our findings suggesting divergent functions for Tfh subsets and the similarity in function of CD4+ Tfh-like CD103+ CTL and exhausted CD8+ CTL.

Finally, we investigated the clinical relevance of the Tfh-like cell states identified in this study, across large breast cancer cohorts. Gene expression signatures were derived for each Tfh-like subset based on their top differentially expressed genes, with CD8+ exhausted (Tex) and CD4+ Treg clusters included in the visualisation for reference (**Extended Data Fig. 6A**). The C06b CD103+ Tfh-like cell signature was significantly enriched in TNBC, compared to other clinical subtypes, across three independent breast cancer transcriptomic datasets: METABRIC^77^, TCGA^78^ and SCAN-B^79^ (Figure 6A). In contrast, signatures corresponding to the C06a CXCR4+ and C06c IGFL2+ Tfh-like subsets were only modestly and inconsistently elevated in TNBC (Figure 6A).

As Tfh-like cells are one of the highest expressors of PD1, we reasoned that their activity may be influenced by therapies targeting PD1. We examined a scRNA-Seq dataset that explored the effect of anti-PD-1 checkpoint inhibitors on human breast cancers^4^. In this study T cell clonal expansion was used as a surrogate measure of responsiveness and anti-tumor activity levels, with patients stratified as T cell ‘expanders’ vs ‘non-expanders’. We used our gene signatures to characterise C06b Tfh: CD103 (differentiated via LAG3 in this dataset) and C06c Tfh: IGFL2 phenotype cells in this dataset (**Extended Data Fig. 6B**). The baseline proportion of both Tfh-like cell subsets was substantially higher in patients classified as expanders, versus those that did not, and the C06b Tfh: CD103+ (LAG3) cells showed a more pronounced association with response, even greater than exhausted CD8 T cells, which are the presumptive target of anti-PD1 treatment (Figure 6B). When we examined changes in Tfh subset abundance during treatment, stratified by clinical response, we observed a significant increase in the ratio of C06b CD103 (LAG3) Tfh-like cells to C06c IGFL2 Tfh-like cells during anti–PD-1 therapy in expanders, but not in non-expanders (Figure 6C; **Extended Data Fig. 6C**). This observation prompted us to evaluate the impact of PD-1 blockade across all T-cell subsets, in order to contextualize Tfh-like dynamics relative to other T-cell populations (Figure 6D; **Extended Data Fig. 6D–H**).

Using patient-matched pre-treatment and on-treatment samples, we observed heterogeneous changes in cell-type proportions across the T-cell compartment (**Extended Data Fig. 6D-F**). Notably, whereas C06b CD103+ (LAG3) Tfh-like cells preferentially expanded in responders on treatment, the C06c IGFL2+ Tfh-like subset increased preferably in non-expanders (**Extended Data Fig. 6E–F**).

Consistent with these abundance shifts, differential gene expression analysis comparing pre- and on-treatment samples revealed marked divergence between expanders and non-expanders across T-cell subsets (Figure 6D; **Extended Data Fig. 6G**). Among all T-cell populations, C06b CD103+ Tfh-like cells exhibited the largest transcriptional response to anti PD-1 therapy in expanders, with minimal changes observed in non-expanders (Figure 6D; **Extended Data Fig. 6G**). Overall, C06b CD103+ Tfh-like cells displayed approximately sixfold more differentially expressed genes in expanders than in non-expanders (**Extended Data Fig. 6H**). In contrast, C06c IGFL2 Tfh-like cells showed comparatively increased transcriptional modulation in non-expanders. Despite these differences, both Tfh-like subsets shared induction of a subset of genes following PD-1 blockade irrespective of response status, such as TXNIP or ZFP36L1 (Figure 6E). However, other genes exhibited response-specific regulation: for example, the chemokine CCL4 was upregulated more towards non-expanders within the C06b CD103+ (Tfh_LAG3) Tfh-like subset, whereas its induction was more pronounced in expanders within the C06c Tfh IGFL2+ subset (Figure 6E). We additionally explored the repertoire diversity amongst T-cells subtypes (Figure 6F–G). Consistent with our pseudotime analysis and with other studies suggesting a common progenitor for Tfh and cytotoxic CD4+ cells^60,63^, TCR sharing analysis demonstrated both C06b and C06c Tfh-like phenotypes to be clonally related (Fig 6G). Moreover, C06b CD103 Tfh were the second most expanded T-cell type in response to anti-PD1 treatment (62.4%), with only CD8 Exhausted more expanded (73.%) (Figure 6F). In contrast, we find only 35.5% of C06c Tfh:IGFL2 cells to be expanded. Therefore, although Tfh cells as a broad classification have been proposed as biomarkers of active anti-cancer immunity, we find a specific subpopulation of these cells associated with improved response to immunotherapy.

## Discussion

Immunological classification has historically relied on a limited set of cell surface protein measurements; however, the advent of single-cell RNA sequencing has significantly expanded our understanding of the diverse functional cell types and states of the immune system^25,34–36^. Integrated proteogenomic frameworks, such as the one presented in this study, improve phenotype resolution and allow precise linking of cellular transcriptomics to classical protein-based cell type dictionaries, such as those generated by the Immunological Genome Project^80^. Our findings demonstrate that this integrative approach can elucidate the functional implications of immune cell gene expression within the tumor microenvironment (TME), effectively bridging classical immunological literature and functional assays with transcriptomic atlases. For example, we identify CD49f as a robust marker of naïve-like or recently activated T cells, and CD103 as primary a marker of T cells exhibiting transcriptional profiles characteristic of exhaustion, preferentially localized at the tumor interface.

Using integrated RNA and ADT analysis, we identified cell types and states that were not discernible through transcriptomics alone (Figures 1–2)^6,7,24,37–39^. This approach offers clear advantages in settings where only modest numbers of cells can be captured, a frequent constraint in clinical trial derived samples. Notably, integrated analysis proved especially effective in characterizing cells with low transcriptional activity such as innate lymphoid cells (ILCs) and γδ T cells, which lack distinct gene expression modules but express specific protein markers. In studies that focus on breast cancer^4,6–8,24^ (including this one), 3’ captured gene expression data alone was insufficient to distinguish MAIT cells from other cytotoxic T lymphocytes (CTLs) (Figure 2F; **Extended Data Fig. D-F**). Given the emerging role of unconventional T cells and ILCs in modulating tumor immunity^45,81^, refining their identification through integrated proteogenomics may prove extremely valuable.

CITE-Seq on dissociated tumors is technically difficult, often yielding low signal-to-noise for low-expression proteins. This limitation hampers unsupervised clustering, especially for cell types that express few markers. Droplet-based platforms also capture fewer cells, underrepresent some types (e.g., neutrophils), and lack granularity/size data, so they cannot fully replace flow cytometry. Instead, ADT-derived cytometry serves as a complementary tool for discovery, guiding targeted flow cytometric analysis. Our marker decision tree (Figure. 2G) is a step toward optimized flow-based identification.

The canonical role of Tfh cells (alternately called “Tph” outside lymphoid organs) is to support B cell differentiation in germinal centers, yet we find approximately twice as many “Tfh-like” cells (PD1+ CXCL13+ BCL6+) as B cells in this breast cancer scRNA-Seq dataset (B cells = 222, Tfh-like cells = 483), raising questions about their function (Figure 1). In the TME, Tfh-like cells express the highest levels of PD-1, the target of the most widespread checkpoint immunotherapy currently in the clinic. Within the breast cancer TME, cells classified as Tfh cells by RNA-seq alone are resolved into three distinct states by proteotranscriptomic analysis. We show that they have different functional gene expression features, spatial localization and unique predicted signalling interactions with neighbouring cells. Our Tfh-like trajectory analysis supports a model of lineage bifurcation where CXCR4^high^/BCL6^high^/ IL7R^high^ progenitor (C06a) differentiates into two functional distinct fates. The first path corresponds to an IGFL2^high^/NMB^high^/TCF7^high^/BTLA4 ^high^ population (C06c), found with elevated protein expression of CD40LG and HLA-DR, that resides predominantly within lymphoid aggregates and spatially associates with B cells, a phenotype similar to T peripheral helper cells (Tph) previously described in both cancers and autoimmune states^82^. The second branch (C06b) becomes a population with distinct expression of CD103 and 2B4, and displaying an exhausted-like state *HAVCR2* (*TIM3*)^high^/*LAG3*^high^, as well as markers of tissue residency and cytotoxicity, markers typically associated with CD8+ Trm and Tex^6,83^. The relatedness between Tfh-like subsets is further supported by our repertoire analysis of data from the Bassez et al. clinical cohort^4^, where we find that IGFL2 Tfh and CD103 Tfh (LAG3 in this dataset) share a large fraction of TCRs (Figure 6D). Complementing of our analysis, mouse model studies have shown that CD4+ non-cytotoxic Tfh-like TIL are progenitors for terminally differentiated cytotoxic CD4+ cells^60^.

Interestingly, in our dataset, C06b CD103+ *LAG3*+ Tfh-like cells and CD103+ *LAG3+* exhausted CD8 T cells expressed highly overlapping RNA signatures and protein markers (Figure 3). GSEA revealed that these two subsets are functionally more similar to each other than to any other T-cell subsets, despite originating from CD4 and CD8 lineages respectively (Figure 3D, **Extended Data Figure 2P**). The expression profile of C06b CD103+ Tfh-like cells also overlaps with that reported for cytotoxic CD4+ T cells^58,59^. Although the origins of CD4+ CTL are unclear^15^, our data add weight to evidence that a common differentiation pathway gives rise to (1) Tfh-like cells that participate in B cell help and (2) CD4+ CTL^63^. CD4 CTL can potentiate direct cancer killing in numerous settings^15^. Indeed, Zhou et al. recently demonstrated that mouse Tfh-derived CD4+CTL resembling our C06b CD103+ Tfh subset can kill MHC-lI+ tumors^60^. A common developmental pathway resulting in Tfh-like cells as well as the CD4+, cytotoxic, CXCL13+PD1+CD103+LAG3+ phenotype may underpin the TLS association with response to checkpoint immunotherapy.

Tfh-like functional divergence is also reflected in spatial transcriptomics, where the subtypes had distinct ecological niches within the tumour. CD103+ Tfh-like cells, together with CD103+ CD8+ T cells, were frequently positioned at the tumour-immune interface, spatially distant from B cells (Figure 4 & 5). Notably, we found both CD4+ and CD8+ CD103+ T co-localised with CXCL10 macrophages, as was noted in other cancers, albeit at a lower spatial profiling resolution^75,76^. However, we observed CD103+ T and CXCL10 Macrophage co-localisation even when distant from the tumour border (Figure 5F–G). Integrating this spatial topology with their shared expression of PD1, CXCL13, CCL chemokines and CSF1, alongside high expression of PDL1 levels by CXCL10 macrophages, our ligand-receptor modeling implies a potential paracrine loop. We propose that CD103+ T cells across both CD4+ and CD8+ lineages may recruit or sustain inflammatory CXCL10+ macrophages, potentially coordinating an antitumor program that extends beyond direct cytotoxicity. These findings are consistent with emerging mouse data indicating that exhausted T-cell populations can retain effector potential and that CD4^+^ Tfh-like subsets may act outside their canonical B-cell helping role^84^. Conversely, IGFL2+ Tfh cells localise to tertiary lymphoid structures, consistent with a conventional helper function, resembling Tph cells reported by Ma et al.^82^ to support B-cell differentiation. However, as we utilized TCF7+ as a spatial surrogate for this non-cytotoxic/exhausted state, this distinct spatial niche likely encompasses both the IGFL2+ subset and the TCF7+ CXCR4+ progenitor pool.

Our data highlights the importance of demarcating heterogeneity within the Tfh-like population when investigating associations with immunotherapeutic efficacy. In the reanalysis of longitudinal TNBC cohort of Bassez et al.^4^, we found the ratio of cytotoxic CD103+(LAG3+) Tfh-like cells (C06b) to helper IGFL2+ Tfh-like cells (C06c) was more strongly associated with response than total Tfh abundance (Figure 6). This suggests that the balance between these divergent states - one engaging cancer cells and the other supporting the lymphoid niche, may influence clinical outcome. We found the enrichment of CD103+ CD4+ Tfh-like cells were more strongly associated with treatment response than CD8+ Tex cells, underscoring the functional relevance of this CD4+ effector program. When evaluating transcriptional change and enrichment of all T-cell subsets in expanders against non-expanders, prior to and during treatment (Figure 6D; **Extended Data Figure 6 D-H**), CD103+(LAG3+) Tfh-like cells emerged as the T-cell subset most selectively impacted by PD-1 blockade with effects that depended on patient expansion status.

*CXCL13*+ T-cell populations clonally expand with immune checkpoint inhibitor treatment^4^. In melanoma, tumor antigen reactive CD4+ cells were restricted to the CXCL13+PD1^high^ fraction^57^ and could be divided into several states that resemble the populations we observed here (ie. Cytotoxic CD103+LAG3+IFNg+ and Tfh memory like TCF7+, Bcl6+, CXCR5+ cells)^57^. These data suggest the CXCL13+Tfh-like TIL contain tumor reactive cells. In support of their clinical association, we observe that the expression signature of the C06b CD103+ Tfh-like cells is enriched in TNBC, which is the breast cancer subtype with the highest mutational burden and the best response rates to immunotherapy. Indeed these cells might be important for direct tumour killing, a proposition strongly supported by murine modelling demonstrating that cytotoxic, exhausted, Tfh-derived CD4+ cells are able to directly kill MHC-lI+ tumor cells^60^. In that study, efficacy of this subset in vivo required both anti-PD1 (acting on Tfh-like precursors to expand progenitor CD4+ CTL) and anti-LAG3 to relieve exhaustion^60^.

In summary, our study illustrates that integrated proteogenomic profiling resolves functional cell states in complex tissue environments with a precision unattainable by unimodal approaches. Leveraging this resolution, we identified a robust biomarker of immunotherapeutic response that retains predictive power even in low-depth and low-cell transcriptomic datasets. Biologically, we re-define the Tfh-like compartment not merely as B-cell helpers, but as a critical progenitor reservoir for CD4+ effector populations within the tumor (summarized in Fig. 6F). Given that macrophage infiltration and T-cell exhaustion are established prognostic determinants^9,85^ our characterization of a spatially coordinated CXCL10+ macrophage–Tfh interaction axis provides a mechanistic basis for these clinical observations, offering new precision targets for therapeutic intervention.

## Methods

### Patient material, ethics approval and consent for publication

The human breast cancer samples used in this study were collected following protocols x13–0133, x16–018, x17–155, x19–0496. Ethical approval for this study was acquired by the Sydney Local Health Districts Ethics committee, St Vincent’s hospital Ethics Committee, and Royal Prince Alfred Hospital zone. Consent for the use of samples in this study was obtained from all patients prior to collection of tissue, and data were de-identified as per approved protocol.

### Single-cell suspension generation of samples

Breast tissue was enzymatically and mechanically dissociated as per the Human Tumor Dissociation Kit (Miltenyi Biotec) protocol. The dissociated breast sample was then passed through a 100 μm MACS Smart Strainers (Miltenyi Biotec), topped up with RPMI 1640 10% FCS then centrifuged at 300 × g for 5 min. Supernatant was discarded, red blood cells were lysed using RBC lysing buffer (Becton Dickinson) for 5 minutes, then washed twice in PBS 10% FCS. All samples were cryopreserved in 10% DMSO, 40% RPMI 1640 and 50 % FBS solution then stored in liquid nitrogen until day of experiment, when samples were thawed in a 37°C liquid bath for 2 minutes, washed twice in RPMI 1640 10% FBS media, passed through a 100 μm strainer then resuspended in 100ul PBS 10% FCS media.

### Flow cytometry and Fluorescence-Activated Cell Sorting (FACS)

Cell suspensions for flow cytometry or FACS were maintained in PBS 2.5% FBS, transferred into appropriate wells of a 96-well U bottom plate. To prevent non-specific antibody binding, cells were incubated with Fc blocking antibody for 20 min at 4C, in the dark. Cells were then incubated with fluorescently-labelled antibodies (**Table S7**) for 25–30 min, on ice and in the dark. Where relevant, cells were fixed in 10% formalin (Sigma-Aldrich) for 15 min at 4 C, and washed and resuspended in PBS. Stained single-cell suspensions were acquired on the BD LSRFortessa^™^. Where relevant, for live (non-fixed) cells, immune populations were sorted by FACS on a FACS Aria III (BD Biosciences).

### Sample preparation and CITE-Seq antibody staining

TotalSeq-A antibodies (Biolegend, USA) compatible with 10X Chromium 3’ mRNA platform were used. The list of antibodies used for each sample are provided in the Supplement (**Table S1**). CITE-Seq was performed as previously described by Stoeckius et. al^86^ with the following modifications: Approximately 1 million cells per sample were resuspended in 95 ul of cell staining buffer (Biolegend, USA) with 5 ul of Fc receptor Block (TrueStain FcX, Biolegend, USA) for 15 min. Cells were then centrifuged at 350 × g for 5 min, supernatant discard, then 100ul of CITE-Seq mastermix (0.5ug of each Antibody) which was prepared earlier in that day with staining buffer (Biolegend, USA) was added to palleted samples. Cells were incubated for 30 min on ice, then washed three times. Approximately 3% 3T3 Mouse cells were then spiked into each sample as control, to estimate ambient RNA and ADT.

### Single-cell capture using 10x genomics chromium and sequencing

Cells for each sample were counted and confirmed to have > 80% viability using haemocytometer. Recovery of a total of 4000 to 6000 cells was the aim for each sample. Single-cell captures were performed using 10X Chromium Single-Cell 3’ v3 with exception to one breast tissue sample where Single-cell 3’ v2 kit was used (**Table S1**). Sample CID4676, CID4660, CID4664 were captured as one pool (multiplexed). Manufacturers protocol was followed in the preparation of RNA and ADT cDNA libraries. The cDNA libraries generated for each respective modality were sequenced separately on Illumina NextSeq 500. The following cycle settings were used for RNA cDNA libraries 28bp (Read 1), 91bp (Read 2) and 8bp (Index) and we aimed for 50,000 reads per cell. The following cycle settings were used for ADT cDNA libraries 28bp (Read 1), 24bp (Read 2) and 8bp (Index) and we aimed for 35,000 reads per cell.

### Single-cell RNA data processing

10x Genomics Cell Ranger (v3.0.4) was used to demultiplex BCL files to FASTQs, cell barcode demultiplexing, genome reference alignment (GRCh38 and mm10) towards generation of unique molecule identifier (UMI) count matrices. The CellRanger UMI counts from the “filtered barcode” list were used. All cells that have greater than 25% mitochondrial content and/or between 10% and 95% mouse mm10 aligned UMI’s were discarded as doublets, low quality cells, or as cells with increased ambient contamination. All mouse UMI counts except those expressed by the top 100 genes were removed prior to analysis. Samples CID4676, CID4660, CID4664 were demultiplexed using method “Souporcell”^87^ as instructed by developers and using default parameters. Genotype information for demultiplexing was generated by running UK Biobank Axiom array (ThermoFisher Scientific, Catalogue 902502) on each patient PBMC. The R package Seurat v4.0.4 was used for normalising, scaling, dimensionality reduction and cluster assignment using default parameters with two deviations, the first 40 principle components (PC) were used for dimensional reduction and to generate nearest neighbour graph, and an increased resolution of ‘1’ was used for clustering. Sample CID4676 was removed from all downstream analysis as it contained less than 50 cells.

### CITE-Seq data processing

The FASTQ demultiplexed reads for ADT libraries were assigned to each cell and antibody using package CITE-Seq count (v1.4.3, https://github.com/Hoohm/CITE-seq-Count), using the authors’ recommended parameters. Briefly, a cell barcode whitelist obtained from Cell Ranger “filtered” out for each sample was used for cell demultiplexing. A cell barcode levenshtein distance of 1 (--bc_collapsing_dist 1) and UMI distance of 2 (--umi_collapsing_dist 2) was allowed to be collapsed. The Antibody barcode list for TotalSeq-A (Biolegend, USA) used to demultiplex ADT is provided in **Table S1**. A levenshtein distance of 3 was permitted for ADT barcode demultiplexing (--max-error 3).

ADT counts were normalised using Seurat v4’s inbuilt centered-log ratio (CLR) transformation within cells (Margin 2). To determine which antibodies were enriched, we first constructed a nearest neighbour graph (‘FindNeigbours”), clustered cells on RNA (‘FindClusters’) at 1.2 resolution, the median absolute deviation of cells from each cluster was calculated and any cells that had 10-fold total ADT counts were discarded. To determine which antibodies are enriched within each sample, Seurat’s ‘FindAllMarkers’ across all clusters at 1.2 resolution after excluding mouse cells, was run for each individual sample. Only ADT features found to be enriched were used in the PC analysis, from which then the first 20 PCs were used towards dimensional reduction, graph construction and clustering analysis.

### Batch correction and Integration of RNA and CITE-Seq data

RNA and ADT assays were both first batch corrected across patients within each respective assay using Seurat v4 (4.0.4) prior to integration across assays. For RNA, the default parameters were used with the following deviation: the top 5000 anchor features were used in the step “FindIntegrationAnchors”, and the top 40 PC dimensions were used for the “IntegrateData” step. The top 40 PCs were similarly used for all subsequent steps; nearest neighbourhood calculation, cluster determination, UMAP calculation. ADT assay was processed similarly to RNA however only the top 20 PCs were used, and all ADT features (except Isotype controls) were used as anchors. The Batch corrected RNA and ADT matrices were then integrated using SeuratV4’s weighted-nearest neighbour (WNN), an approach which allows for simultaneous clustering of cells based on RNA and surface protein expression (ADT)^33^. Integration was performed with developer recommended (default) parameters (*k = 20*) with the following modifications: The first 50 PC dimensions were used for RNA, and the first 20 PCs for ADT, in step “FindMultiModalNeighbors”. A resolution of 3.2 and algorithm 3 (SLM - smart local moving) was used for “FindClusters”. The majority of clusters were found to be present in most or all samples, with the exception of neoplastic epithelial cell clusters (C47–51) and clusters containing fewer than 50 cells, such as cluster C32-Mast:TPSB2 ADT-cKIT and C33-Endo:SEMA3G ADT-4.1BBL (**Extended Data Fig. 1C**).

### Silhouette score and cluster probability calculation

The silhouette coefficient was calculated using R package cluster 2.1.0. An euclidean distance matrix was generated from the first 40 PCs calculated from RNA assay alone, the same PCs which were used for dimensionality reduction and clustering as described above for processing RNA counts. The cell annotations were derived from the WNN approach, calculated from RNA and ADT assays as described above and shown for all cell lineages as in Figure 1 or targeted TIL analysis in Figure 2. To calculate the cluster probability, we used an approach previously described by Lun et al. (^88^ Scran https://bioconductor.org/packages/devel/bioc/vignettes/scran/inst/doc/scran.html) which measures how many cells were partitioned into the same cluster after bootstrapping. We took the PCAs generated by Seurat’s v4 RNA assay analysis however with cell assignments to clusters set as per WNN of ADT and RNA modalities as described above. R Package Scran v1.18 was then used to bootstrap clusters (‘*bootstrapCluster*’) and to generate shared nearest neighbour graphs, and the paired co-assignment probability of cells to the same partition was evaluated using package igraph v1.2.6 function ‘cluster_walktrap’.

### Differential expression analysis, gene signature score modules, GO enrichment analysis.

Differential gene expression analysis was performed using R package Seurat v4.0.4 function ‘FindAllMarkers’, using MAST v1.16.0 test^89^. Module scoring for each gene signature was calculated using Seurat’s ‘AddModulScore’. The list of signatures used and their source are available in **Table S4**. The cluster median module score of each signature was scaled 0–1 then visualised using spider plots using R package fmsb v0.7.0 (CRAN). R Package VISION v2.1 (DeTomaso et al., 2019) was employed to calculate enrichment of Gene ontology (GO) biological process’s, using immunological signature gene sets (c7.all.v7.2.symbols.gmt) derived from Molecular Signatures Database (MSigDB)^90^. Either Complex Heatmap v2.7.11^22^ or pheatmap v1.0.12 (CRAN) were used to visualise differentially expressed ADT, RNA, module scores and GO results. Seurat’s ‘DotPlot” function was used to visualise all dot plots. R package AUCell v1.12^91^ was used to score CD8 T-cell Bulk RNA-seq signatures from Savas et al^6^.

### Visium transcriptomic analysis

Visium patient sample counts and pathology notes were sourced from Wu et al. study^7^. The single-cell dataset used towards deconvolution was similarly taken from Wu et al. study^7^ however further processed to stratify Tfh cluster into CD103 Tfh and IGFL2 Tfh. To integrate the single-cell and spatial transcriptomics data (Visium), we used the software *stereoscope* v. 0.3.1^92^. As input, the method takes raw UMI count data from the single cell and spatial transcriptomics experiments, together with cell type annotations for the former. From this, a single proportion matrix is produced. The matrix gives the proportion of each cell type (defined in the single cell data) at every spatial location. To improve the performance of stereoscope, we used a curated set of highly variable genes.

In order to reduce the runtime, we employed a subsampling strategy similar to that proposed in the original *stereoscope* manuscript. More specifically, we first defined a lower and upper bound (here, 25 and 250 respectively). Next, cells were sampled according to the following scheme: if a cell type had fewer members than the lower bound, we excluded it from the analysis; if a cell type had more or the same number of members as the lower bound, but fewer or the same number of members as the upper bound, we used all cells within the cell type; if a cell type had more members than the upper bound, we randomly sampled #[upper bound] cells from the cell type (without replacement).

*stereoscope* was run with the following parameter settings: batch size - 2048, number of epochs - 50000, These settings were used in both steps of *stereoscope*, i.e., the parameter inference step and the proportion estimation step. Default values were used for all other parameters.

Highly variable genes were extracted by applying a sequence of three functions from the scanpy suite (v. 1.7.2) to the single cell data. First, we normalized the gene expression data, then, the normalized values were log-transformed (using pseudocount 1), finally, the highly variable genes were identified from the transformed values. The exact function calls were:

scanpy.pp.normalize_per_cell(…,10e4)

scanpy.pp.log1p(…)

scanpy.pp.highly_variable_genes(…,n_top_genes=5000)

Where “…” represents an anndata object containing the relevant data.

### Trajectory analysis & Receptor-Ligand analysis

R package Monocle3 v 1.0^93^ was used to generate the pseudo-trajectory analysis involved with the characterisation of T follicular helper cells. Briefly, the RNA batch corrected and integrated matrix generated by Seurat v4 (4.0.4) were exported to build the CellDataSet object. Pseudotemporal analysis was then performed using default parameters as instructed by developers in their vignette.

The R package Slingshot v1.6.1^94^ was used to generate the pseudo-trajectory analysis of CD8 T-cells. Default parameters were used, and UMAP input was derived from batch corrected RNA matrix values as generated by Seurat v4 (4.0.4). Trajectory overlay was mapped on cells clustered and annotated by Seurat v4 (4.0.4).

Ligand-Receptor analysis was performed using R package “CellChat”^73^. Analysis was executed using default parameters as recommended by developers, as described by their vignettes.

### TCR clonal expansion and inter-subset sharing analysis

Single-cell TCR and transcriptomic data from Bassez et al^4^. were reclustered to distinguish LAG3+ Tfh (surrogate marker to identify C06b CD103+ signature cells) from IGFL2+ Tfh subsets, then analyzed to quantify clonal expansion and inter-subset TCR sharing. Clonotypes were stratified by cell count into four categories: Single (1), Small (2–5), Medium (6–20), and Large (>20). For each patient and T cell subset, we calculated the proportion of cells in each category and the percentage of all expanded cells (% of T-cells with 2 >= clones). Mean expansion frequencies were computed across patients for each subset and treatment condition.

Inter-subset clonal relationships were quantified using the Jaccard similarity index for all pairwise subset combinations within individual patients. Patient-specific overlap matrices were averaged to generate a mean repertoire-sharing matrix, visualized as heatmaps and chord diagrams with link width proportional to shared clonotype frequency.

### Xenium In Situ spatial transcriptomics analysis

Formalin-fixed paraffin-embedded (FFPE) 5 mm tissue sections were placed on Xenium slides. The tissue was deparaffinized and decrosslinked as described in Xenium Deparaffinization and Decrosslinking Protocol (Demonstrated protocol CG000580). Predesign Human Breast Cancer panel (280genes) and custom gene panel (100genes) were added to the tissue (See **Table S6** for probes). Probes were hybridized to the target RNA, ligated, and enzymatically amplified generating multiple copies for each RNA target as described in Probe Hybridization, Ligation and Amplification user guide (User guide CG000582). Xenium slides were then loaded for imaging and analysis on the Xenium Analyzer instrument following Decoding and Imaging user guide (User guide CG000584). Instrument software version 1.4.2.0 and software analysis version xenium-1.4.0.6 were used.

Cell-feature matrices were loaded into the anndata format and processed using Scanpy (v1.9.8). Raw gene expression counts were normalized and log-transformed. Graph-based clustering results from the Xenium on-board analysis pipeline were used for cell type annotation. Clusters were assigned cell type labels based on marker genes defined in the Human Breast panel. Epithelial clusters were manually refined using marker expression and morphology to distinguish normal from cancer.

Tfh were identified by scoring T cells for Tfh markers (CD4 or CD8, CXCL13, and BCL6) using Scanpy’s ‘score_genes’ function. The top 10% highest-scoring cells per sample were classified as Tfh. Tfh were further subclassified using two methods: (1) Proximity-based, where Tfh were labeled as close or far from B cells based on a 50 μm distance threshold; (2) Microenvironment-based, where Tfh were categorized as cancer-adjacent or lymph-rich depending on whether cancer cells or B cells were more abundant within a 200 μm radius.

Gene expression comparisons between Tfh subgroups were performed by subsetting Tfh cells and renormalizing, log-transforming, and scaling expression counts. Macrophage group comparisons were conducted similarly.

### Patient cell type proportion analysis

R package speckle (0.0.2)^95^ was used to calculate statistical significance in the change of cell type proportion between non-expander to expander. All Beassez et al. patients^4^ which were not assigned either as Expander (E) or Non-expander (NE) were removed from analysis (two patients’ pre-post samples were excluded). Change in composition was visualised by calculating the fraction of each cell type within each patient. A non-parametric t-test (n=29) was used to calculate the p-value of baseline change of composition between Expanders and Non-expanders. See Bassez et al. for further detail on patient cohort^4^.

### Paired Cellular & Transcriptional Dynamics

To dissect response-specific dynamics, we performed a patient paired analysis of pre- and on-treatment samples. For each patient, the fold change (FC) in T-cell subset frequency was calculated as log2(On/Pre). Patients with incomplete timepoints were excluded to ensure robust paired testing. Expansion bias was quantified as the difference in mean FC between clinical responders (Expanders) and non-responders (Delta = Expander mean - Non-Expander mean), with significance assessed via Wilcoxon rank-sum tests. Differential gene expression (DGE) was computed for each cluster comparing On- vs. Pre-treatment conditions within each response group using Seurat’s FindMarkers (Wilcoxon test). Significant perturbation genes (DEGs) were defined by adjusted P < 0.05 and absolute log2 FC > 0.25. To evaluate mechanistic similarity, we correlated the log2 FC of Expanders vs. Non-Expanders for each cluster. Genes were classified as “Shared” (significant/concordant in both), “Expander-Specific,” or “Non-Expander-Specific”. The ratio of Tfh-LAG3 to Tfh-IGFL2 abundance was calculated per patient. Delta ratio represents the absolute change (On minus Pre); unified comparisons utilized Wilcoxon tests to assess group-level differences.

### Statistics and reproducibility

No statistical method was used to predetermine sample size. The statistical significance for all differentially expressed genes or ADT were determined using MAST^89^, and adjusted bonferroni corrected values were used. The Box plot centre line depicts the median value, the first and third line mark the 25% and 75% quantile, the whiskers correspond to 1.5x the interquartile range (IQR), and dots mark outliers. Details for any statistical tests performed are present in figure legends and in relevant method sections.

## Supplementary Material

Supplementary Files

This is a list of supplementary les associated with this preprint. Click to download.

- SupplementarytableS1CITEpanelADT.xlsx
- SupplementarytableS2patientSampleInfo.xlsx
- SupplementarytableS5Correlationprotein.csv
- SupplementaryTableS8flowantibodies.xlsx
- SupplementarytableS4Otherstudygenesignatures.csv
- SupplementarytableS6Xeniumpanel.xlsx
- SupplementarytableS3DGEout.xlsx
- CombinedExtendedDataFigures2.pdf
- SupplementarytableS7Tfhsurvivalgenes.xlsx

## Data Availability

All scripts used to process data and perform statistical analysis are available on https://github.com/Swarbricklab-code/BrCa_Integrated_proteogenomics. Raw FASTQ data can be accessed from the NCBI Gene Expression Omnibus database GSE199219. Xenium datasets are uploaded to Zenodo pt 3946, pt 3962, pt 4754. and Any code used to visualise data is available from the corresponding authors upon reasonable request.

## References

[1] Hanahan D. & Weinberg R. A. Hallmarks of cancer: the next generation. Cell 144, 646–674 (2011). 21376230 10.1016/j.cell.2011.02.013

[2] Bruni D., Angell H. K. & Galon J. The immune contexture and Immunoscore in cancer prognosis and therapeutic efficacy. Nat. Rev. Cancer 20, 662–680 (2020). 32753728 10.1038/s41568-020-0285-7

[3] Valkenburg K. C., de Groot A. E. & Pienta K. J. Targeting the tumour stroma to improve cancer therapy. Nat. Rev. Clin. Oncol. 15, 366–381 (2018). 29651130 10.1038/s41571-018-0007-1 PMC5960434

[4] Bassez A. A single-cell map of intratumoral changes during anti-PD1 treatment of patients with breast cancer. Nat. Med. 27, 820–832 (2021). 33958794 10.1038/s41591-021-01323-8

[5] Savas P. Clinical relevance of host immunity in breast cancer: from TILs to the clinic. Nat. Rev. Clin. Oncol. 13, 228–241 (2016). 26667975 10.1038/nrclinonc.2015.215

[6] Savas P. Single-cell profiling of breast cancer T cells reveals a tissue-resident memory subset associated with improved prognosis. Nat. Med. 24, 986–993 (2018). 29942092 10.1038/s41591-018-0078-7

[7] Wu S. Z. A single-cell and spatially resolved atlas of human breast cancers. Nat. Genet. 53, 1334–1347 (2021). 34493872 10.1038/s41588-021-00911-1 PMC9044823

[8] Zhang Y. Single-cell analyses reveal key immune cell subsets associated with response to PD-L1 blockade in triple-negative breast cancer. Cancer Cell 39, 1578–1593.e8 (2021). 34653365 10.1016/j.ccell.2021.09.010

[9] Cassetta L. Human tumor-associated macrophage and monocyte transcriptional landscapes reveal cancer-specific reprogramming, biomarkers, and therapeutic targets. Cancer Cell 35, 588–602.e10 (2019). 30930117 10.1016/j.ccell.2019.02.009 PMC6472943

[10] Molgora M. TREM2 modulation remodels the tumor myeloid landscape enhancing anti-PD-1 immunotherapy. Cell 182, 886–900.e17 (2020). 32783918 10.1016/j.cell.2020.07.013 PMC7485282

[11] Wei S. C., Duffy C. R. & Allison J. P. Fundamental mechanisms of immune checkpoint blockade therapy. Cancer Discov. 8, 1069–1086 (2018). 30115704 10.1158/2159-8290.CD-18-0367

[12] Emens L. A. & Loi S. Immunotherapy approaches for breast cancer patients in 2023. Cold Spring Harb. Perspect. Med. 13, (2023). 10.1101/cshperspect.a041332 PMC10071416 37011999

[13] Rao D. A. Pathologically expanded peripheral T helper cell subset drives B cells in rheumatoid arthritis. Nature 542, 110–114 (2017). 28150777 10.1038/nature20810 PMC5349321

[14] Speiser D. E., Chijioke O., Schaeuble K. & Münz C. CD4+ T cells in cancer. Nat. Cancer 4, 317–329 (2023). 36894637 10.1038/s43018-023-00521-2

[15] Montauti E., Oh D. Y. & Fong L. CD4+ T cells in antitumor immunity. Trends Cancer 10, 969–985 (2024). 39242276 10.1016/j.trecan.2024.07.009 PMC11464182

[16] Poncette L., Bluhm J. & Blankenstein T. The role of CD4 T cells in rejection of solid tumors. Curr. Opin. Immunol. 74, 18–24 (2022). 34619457 10.1016/j.coi.2021.09.005 PMC8933281

[17] Hollern D. P. B cells and T follicular helper cells mediate response to checkpoint inhibitors in high mutation burden mouse models of breast cancer. Cell 179, 1191–1206.e21 (2019). 31730857 10.1016/j.cell.2019.10.028 PMC6911685

[18] Bronsert P. High numbers and densities of PD1+ T-follicular helper cells in triple-negative breast cancer draining lymph nodes are associated with lower survival. Int. J. Mol. Sci. 21, 5948 (2020). 32824917 10.3390/ijms21175948 PMC7504397

[19] Noël G. Functional Th1-oriented T follicular helper cells that infiltrate human breast cancer promote effective adaptive immunity. J. Clin. Invest. 131, (2021). 10.1172/JCI139905 PMC8483751 34411002

[20] Voabil P. An ex vivo tumor fragment platform to dissect response to PD-1 blockade in cancer. Nat. Med. 27, 1250–1261 (2021). 34239134 10.1038/s41591-021-01398-3

[21] Bindea G. Spatiotemporal dynamics of intratumoral immune cells reveal the immune landscape in human cancer. Immunity 39, 782–795 (2013). 24138885 10.1016/j.immuni.2013.10.003

[22] Gu Z., Eils R. & Schlesner M. Complex heatmaps reveal patterns and correlations in multidimensional genomic data. Bioinformatics 32, 2847–2849 (2016). 27207943 10.1093/bioinformatics/btw313

[23] Litchfield K. Meta-analysis of tumor- and T cell-intrinsic mechanisms of sensitization to checkpoint inhibition. Cell 184, 596–614.e14 (2021). 33508232 10.1016/j.cell.2021.01.002 PMC7933824

[24] Azizi E. Single-cell map of diverse immune phenotypes in the breast tumor microenvironment. Cell 174, 1293–1308.e36 (2018). 29961579 10.1016/j.cell.2018.05.060 PMC6348010

[25] Cheng S. A pan-cancer single-cell transcriptional atlas of tumor infiltrating myeloid cells. Cell 184, 792–809.e23 (2021). 33545035 10.1016/j.cell.2021.01.010

[26] La Manno G. RNA velocity of single cells. Nature 560, 494–498 (2018). 30089906 10.1038/s41586-018-0414-6 PMC6130801

[27] Svensson V., Vento-Tormo R. & Teichmann S. A. Exponential scaling of single-cell RNA-seq in the past decade. Nat. Protoc. 13, 599–604 (2018). 29494575 10.1038/nprot.2017.149

[28] Akan P. Comprehensive analysis of the genome transcriptome and proteome landscapes of three tumor cell lines. Genome Med. 4, 86 (2012). 23158748 10.1186/gm387 PMC3580420

[29] Buccitelli C. & Selbach M. mRNAs, proteins and the emerging principles of gene expression control. Nat. Rev. Genet. 21, 630–644 (2020). 32709985 10.1038/s41576-020-0258-4

[30] Schwanhäusser B. Global quantification of mammalian gene expression control. Nature 473, 337–342 (2011). 21593866 10.1038/nature10098

[31] Peterson V. M. Multiplexed quantification of proteins and transcripts in single cells. Nat. Biotechnol. 35, 936–939 (2017). 28854175 10.1038/nbt.3973

[32] Stoeckius M. Simultaneous epitope and transcriptome measurement in single cells. Nat. Methods 14, 865–868 (2017). 28759029 10.1038/nmeth.4380 PMC5669064

[33] Hao Y. Integrated analysis of multimodal single-cell data. Cell 184, 3573–3587.e29 (2021). 34062119 10.1016/j.cell.2021.04.048 PMC8238499

[34] Triana S. Single-cell proteo-genomic reference maps of the hematopoietic system enable the purification and massive profiling of precisely defined cell states. Nat. Immunol. 22, 1577–1589 (2021). 34811546 10.1038/s41590-021-01059-0 PMC8642243

[35] Mulder K. Cross-tissue single-cell landscape of human monocytes and macrophages in health and disease. Immunity 54, 1883–1900.e5 (2021). 34331874 10.1016/j.immuni.2021.07.007

[36] Zheng L. Pan-cancer single-cell landscape of tumor-infiltrating T cells. Science 374, abe6474 (2021). 10.1126/science.abe6474 34914499

[37] Janssen A. Γδ T-cell receptors derived from breast cancer-infiltrating T lymphocytes mediate antitumor reactivity. Cancer Immunol. Res. 8, 530–543 (2020). 32019779 10.1158/2326-6066.CIR-19-0513

[38] Ruffell B. Leukocyte composition of human breast cancer. Proc. Natl. Acad. Sci. U. S. A. 109, 2796–2801 (2012). 21825174 10.1073/pnas.1104303108 PMC3287000

[39] Wagner J. A single-cell atlas of the tumor and immune ecosystem of human breast cancer. Cell 177, 1330–1345.e18 (2019). 30982598 10.1016/j.cell.2019.03.005 PMC6526772

[40] Rousseeuw P. J. Silhouettes: A graphical aid to the interpretation and validation of cluster analysis. J. Comput. Appl. Math. 20, 53–65 (1987).

[41] Szabo P. A. Single-cell transcriptomics of human T cells reveals tissue and activation signatures in health and disease. Nat. Commun. 10, 4706 (2019). 31624246 10.1038/s41467-019-12464-3 PMC6797728

[42] Wolf T. Dynamics in protein translation sustaining T cell preparedness. Nat. Immunol. 21, 927–937 (2020). 32632289 10.1038/s41590-020-0714-5 PMC7610365

[43] Cano-Gamez E. Single-cell transcriptomics identifies an effectorness gradient shaping the response of CD4+ T cells to cytokines. Nat. Commun. 11, 1801 (2020). 32286271 10.1038/s41467-020-15543-y PMC7156481

[44] Ma L., Feng Y. & Zhou Z. A close look at current γδ T-cell immunotherapy. Front. Immunol. 14, 1140623 (2023). 37063836 10.3389/fimmu.2023.1140623 PMC10102511

[45] Petley E. V. MAIT cells regulate NK cell-mediated tumor immunity. Nat. Commun. 12, 4746 (2021). 34362900 10.1038/s41467-021-25009-4 PMC8346465

[46] Guo X. Global characterization of T cells in non-small-cell lung cancer by single-cell sequencing. Nat. Med. 24, 978–985 (2018). 29942094 10.1038/s41591-018-0045-3

[47] Wang X., He Y., Zhang Q., Ren X. & Zhang Z. Direct comparative analyses of 10X Genomics Chromium and Smart-seq2. Genomics Proteomics Bioinformatics 19, 253–266 (2021). 33662621 10.1016/j.gpb.2020.02.005 PMC8602399

[48] Cibrián D. & Sánchez-Madrid F. CD69: from activation marker to metabolic gatekeeper. Eur. J. Immunol. 47, 946–953 (2017). 28475283 10.1002/eji.201646837 PMC6485631

[49] Kok L., Masopust D. & Schumacher T. N. The precursors of CD8+ tissue resident memory T cells: from lymphoid organs to infected tissues. Nat. Rev. Immunol. 22, 283–293 (2022). 34480118 10.1038/s41577-021-00590-3 PMC8415193

[50] Okła K., Farber D. L. & Zou W. Tissue-resident memory T cells in tumor immunity and immunotherapy. J. Exp. Med. 218, (2021). 10.1084/jem.20201605 PMC7992502 33755718

[51] McArdel S. L., Terhorst C. & Sharpe A. H. Roles of CD48 in regulating immunity and tolerance. Clin. Immunol. 164, 10–20 (2016). 26794910 10.1016/j.clim.2016.01.008 PMC4860950

[52] Binder C. CD2 immunobiology. Front. Immunol. 11, 1090 (2020). 32582179 10.3389/fimmu.2020.01090 PMC7295915

[53] Li H. Dysfunctional CD8 T cells form a proliferative, dynamically regulated compartment within human melanoma. Cell 176, 775–789.e18 (2019). 30595452 10.1016/j.cell.2018.11.043 PMC7253294

[54] Gavil N. V., Cheng K. & Masopust D. Resident memory T cells and cancer. Immunity 57, 1734–1751 (2024). 39142275 10.1016/j.immuni.2024.06.017 PMC11529779

[55] Mackay L. K. Hobit and Blimp1 instruct a universal transcriptional program of tissue residency in lymphocytes. Science 352, 459–463 (2016). 27102484 10.1126/science.aad2035

[56] Zhu S., Lin J., Qiao G., Wang X. & Xu Y. Tim-3 identifies exhausted follicular helper T cells in breast cancer patients. Immunobiology 221, 986–993 (2016). 27156907 10.1016/j.imbio.2016.04.005

[57] Veatch J. R. Neoantigen-specific CD4+ T cells in human melanoma have diverse differentiation states and correlate with CD8+ T cell, macrophage, and B cell function. Cancer Cell 40, 393–409.e9 (2022). 35413271 10.1016/j.ccell.2022.03.006 PMC9011147

[58] Seki N. Cytotoxic Tph subset with low B-cell helper functions and its involvement in systemic lupus erythematosus. Commun. Biol. 7, 277 (2024). 38448723 10.1038/s42003-024-05989-x PMC10918188

[59] Oh D. Y. & Fong L. Cytotoxic CD4+ T cells in cancer: Expanding the immune effector toolbox. Immunity 54, 2701–2711 (2021). 34910940 10.1016/j.immuni.2021.11.015 PMC8809482

[60] Zhou W. Stem-like progenitor and terminally differentiated TFH-like CD4+ T cell exhaustion in the tumor microenvironment. Cell Rep. 43, 113797 (2024). 38363680 10.1016/j.celrep.2024.113797

[61] Emtage P. IGFL: A secreted family with conserved cysteine residues and similarities to the IGF superfamily. Genomics 88, 513–520 (2006). 16890402 10.1016/j.ygeno.2006.05.012

[62] Lobito A. A. Murine insulin growth factor-like (IGFL) and human IGFL1 proteins are induced in inflammatory skin conditions and bind to a novel tumor necrosis factor receptor family member, IGFLR1. J. Biol. Chem. 286, 18969–18981 (2011). 21454693 10.1074/jbc.M111.224626 PMC3099712

[63] Donnarumma T. Opposing development of cytotoxic and follicular helper CD4 T cells controlled by the TCF-1-Bcl6 nexus. Cell Rep. 17, 1571–1583 (2016). 27806296 10.1016/j.celrep.2016.10.013 PMC5149578

[64] Vilgelm A. E. & Richmond A. Chemokines modulate immune surveillance in tumorigenesis, metastasis, and response to immunotherapy. Front. Immunol. 10, 333 (2019). 30873179 10.3389/fimmu.2019.00333 PMC6400988

[65] Morita R. Human blood CXCR5(+)CD4(+) T cells are counterparts of T follicular cells and contain specific subsets that differentially support antibody secretion. Immunity 34, 108–121 (2011). 21215658 10.1016/j.immuni.2010.12.012 PMC3046815

[66] Singh D. Analysis of CXCR5+Th17 cells in relation to disease activity and TNF inhibitor therapy in Rheumatoid Arthritis. Sci. Rep. 6, 39474 (2016). 28004828 10.1038/srep39474 PMC5177940

[67] Cosgrove J. B cell zone reticular cell microenvironments shape CXCL13 gradient formation. Nat. Commun. 11, 3677 (2020). 32699279 10.1038/s41467-020-17135-2 PMC7376062

[68] Havenar-Daughton C. CXCL13 is a plasma biomarker of germinal center activity. Proc. Natl. Acad. Sci. U. S. A. 113, 2702–2707 (2016). 26908875 10.1073/pnas.1520112113 PMC4790995

[69] Mintz M. A. The HVEM-BTLA axis restrains T cell help to germinal center B cells and functions as a cell-extrinsic suppressor in lymphomagenesis. Immunity 51, 310–323.e7 (2019). 31204070 10.1016/j.immuni.2019.05.022 PMC6703922

[70] Xin G. Single-cell RNA sequencing unveils an IL-10-producing helper subset that sustains humoral immunity during persistent infection. Nat. Commun. 9, 5037 (2018). 30487586 10.1038/s41467-018-07492-4 PMC6261948

[71] Ginhoux F., Guilliams M. & Merad M. Expanding dendritic cell nomenclature in the single-cell era. Nat. Rev. Immunol. 22, 67–68 (2022). 35027741 10.1038/s41577-022-00675-7

[72] Maier B. A conserved dendritic-cell regulatory program limits antitumour immunity. Nature 580, 257–262 (2020). 32269339 10.1038/s41586-020-2134-y PMC7787191

[73] Jin S. Inference and analysis of cell-cell communication using CellChat. Nat. Commun. 12, 1088 (2021). 33597522 10.1038/s41467-021-21246-9 PMC7889871

[74] Elewaut A. Cancer cells impair monocyte-mediated T cell stimulation to evade immunity. Nature 637, 716–725 (2025). 39604727 10.1038/s41586-024-08257-4 PMC7617236

[75] Pelka K. Spatially organized multicellular immune hubs in human colorectal cancer. Cell 184, 4734–4752.e20 (2021). 34450029 10.1016/j.cell.2021.08.003 PMC8772395

[76] Chen J. H. Human lung cancer harbors spatially organized stem-immunity hubs associated with response to immunotherapy. Nat. Immunol. 25, 644–658 (2024). 38503922 10.1038/s41590-024-01792-2 PMC12096941

[77] Curtis C. The genomic and transcriptomic architecture of 2,000 breast tumours reveals novel subgroups. Nature 486, 346–352 (2012). 22522925 10.1038/nature10983 PMC3440846

[78] The Cancer Genome Atlas Program (TCGA). https://www.cancer.gov/tcga (2022).

[79] Staaf J. RNA sequencing-based single sample predictors of molecular subtype and risk of recurrence for clinical assessment of early-stage breast cancer. NPJ Breast Cancer 8, 94 (2022). 35974007 10.1038/s41523-022-00465-3 PMC9381586

[80] Immunological Genome Project. ImmGen at 15. Nat. Immunol. 21, 700–703 (2020). 32577013 10.1038/s41590-020-0687-4

[81] Heinrich B. The tumour microenvironment shapes innate lymphoid cells in patients with hepatocellular carcinoma. Gut 71, 1161–1175 (2022). 34340996 10.1136/gutjnl-2021-325288 PMC8807808

[82] Ma J. A blueprint for tumor-infiltrating B cells across human cancers. Science 384, eadj4857 (2024). 10.1126/science.adj4857 38696569

[83] Scott A. C. TOX is a critical regulator of tumour-specific T cell differentiation. Nature 571, 270–274 (2019). 31207604 10.1038/s41586-019-1324-y PMC7698992

[84] Kersten K. Spatiotemporal co-dependency between macrophages and exhausted CD8^+^T cells in cancer. bioRxiv (2021) doi:10.1101/2021.09.27.461866. PMC9197962 35623342

[85] Foroutan M. The ratio of exhausted to resident infiltrating lymphocytes is prognostic for colorectal cancer patient outcome. Cancer Immunol. Res. 9, 1125–1140 (2021). 34413087 10.1158/2326-6066.CIR-21-0137

[86] Stoeckius M., Stoeckius M. & Smibert P. CITE-seq. Protoc. Exch. (2017) doi:10.1038/protex.2017.068.

[87] Heaton H. Souporcell: robust clustering of single-cell RNA-seq data by genotype without reference genotypes. Nat. Methods 17, 615–620 (2020). 32366989 10.1038/s41592-020-0820-1 PMC7617080

[88] Lun A. T. L., McCarthy D. J. & Marioni J. C. A step-by-step workflow for low-level analysis of single-cell RNA-seq data with Bioconductor. F1000Res. 5, 2122 (2016). 27909575 10.12688/f1000research.9501.1 PMC5112579

[89] Finak G. MAST: a flexible statistical framework for assessing transcriptional changes and characterizing heterogeneity in single-cell RNA sequencing data. Genome Biol. 16, 278 (2015). 26653891 10.1186/s13059-015-0844-5 PMC4676162

[90] Subramanian A. Gene set enrichment analysis: a knowledge-based approach for interpreting genome-wide expression profiles. Proc. Natl. Acad. Sci. U. S. A. 102, 15545–15550 (2005). 16199517 10.1073/pnas.0506580102 PMC1239896

[91] Aibar S. SCENIC: single-cell regulatory network inference and clustering. Nat. Methods 14, 1083–1086 (2017). 28991892 10.1038/nmeth.4463 PMC5937676

[92] Andersson A. Single-cell and spatial transcriptomics enables probabilistic inference of cell type topography. Commun. Biol. 3, 565 (2020). 33037292 10.1038/s42003-020-01247-y PMC7547664

[93] Cao J. The single-cell transcriptional landscape of mammalian organogenesis. Nature 566, 496–502 (2019). 30787437 10.1038/s41586-019-0969-x PMC6434952

[94] Street K. Slingshot: cell lineage and pseudotime inference for single-cell transcriptomics. BMC Genomics 19, 477 (2018). 29914354 10.1186/s12864-018-4772-0 PMC6007078

[95] Phipson B. *propeller*: Testing for differences in cell type proportions in single cell data. bioRxiv (2021) doi:10.1101/2021.11.28.470236. PMC9563678 36005887

